# Zinc Finger Binding Motifs Do Not Explain Recombination Rate Variation within or between Species of Drosophila

**DOI:** 10.1371/journal.pone.0045055

**Published:** 2012-09-18

**Authors:** Caiti S. S. Heil, Mohamed A. F. Noor

**Affiliations:** Department of Biology, Duke University, Durham, North Carolina, United States of America; North Carolina State University, United States of America

## Abstract

In humans and mice, the Cys_2_His_2_ zinc finger protein PRDM9 binds to a DNA sequence motif enriched in hotspots of recombination, possibly modifying nucleosomes, and recruiting recombination machinery to initiate Double Strand Breaks (DSBs). However, since its discovery, some researchers have suggested that the recombinational effect of PRDM9 is lineage or species specific. To test for a conserved role of PRDM9-like proteins across taxa, we use the *Drosophila pseudoobscura* species group in an attempt to identify recombination associated zinc finger proteins and motifs. We leveraged the conserved amino acid motifs in Cys_2_His_2_ zinc fingers to predict nucleotide binding motifs for all Cys_2_His_2_ zinc finger proteins in *Drosophila pseudoobscura* and identified associations with empirical measures of recombination rate. Additionally, we utilized recombination maps from *D. pseudoobscura* and *D. miranda* to explore whether changes in the binding motifs between species can account for changes in the recombination landscape, analogous to the effect observed in PRDM9 among human populations. We identified a handful of potential recombination-associated sequence motifs, but the associations are generally tenuous and their biological relevance remains uncertain. Furthermore, we found no evidence that changes in zinc finger DNA binding explains variation in recombination rate between species. We therefore conclude that there is no protein with a DNA sequence specific human-PRDM9-like function in Drosophila. We suggest these findings could be explained by the existence of a different recombination initiation system in Drosophila.

## Introduction

Meiotic recombination is an essential process both mechanistically and evolutionarily, and thus should experience strong selective pressures. However, identifying how selection affects the locations of recombination events is more complex than was once assumed. Recombination rate is variable within and among genomes, displaying significant heterogeneity across most living organisms [1] and evolving rapidly, with recombination “hotspot” turnover in as short as 120,000 years [2]. While years of research have determined some elements associated with recombination rate variation, such as temperature, GC content, repeats, SNP density, chromatin state, and histone modifications, the specific effects of DNA sequence “motifs” have attracted much attention.

The 13-base degenerate motif CCNCCNTNNCCNC is enriched in approximately 40% of European human hotspots, recruiting recombination machinery to initiate double strand breaks [3], [4]. This motif binds the Cys_2_His_2_ zinc finger protein PRDM9 in humans, and allelic variation at *Prdm9* modifies hotspot activity within both humans and mice [3], [5], [6], [7], [8]. The *Prdm9* gene also contains a SET-methyltransferase domain, which is responsible for the common chromatin feature trimethylation of lysine 4 of histone H3, or H3K4me3. H3K4me3 in yeast and mouse seems to be a prominent and pre-existing mark of active recombination sites, creating a link between sequence and epigenetic features affecting recombination [9], [10]. This link inspired a proposed model in humans involving the recognition of a DNA sequence motif by PRDM9 and the modification of adjacent nucleosomes by the SET domain [11]. Proteins with an affinity to the modification H3K4me3 are recruited and may modify the chromatin or nucleosomes further. The conserved topoisomerase II-like protein SPO11 subsequently recognizes one or several of these signals, binds to the DNA at that location, and initiates recombination by a double strand break (DSB).

**Figure 1:**
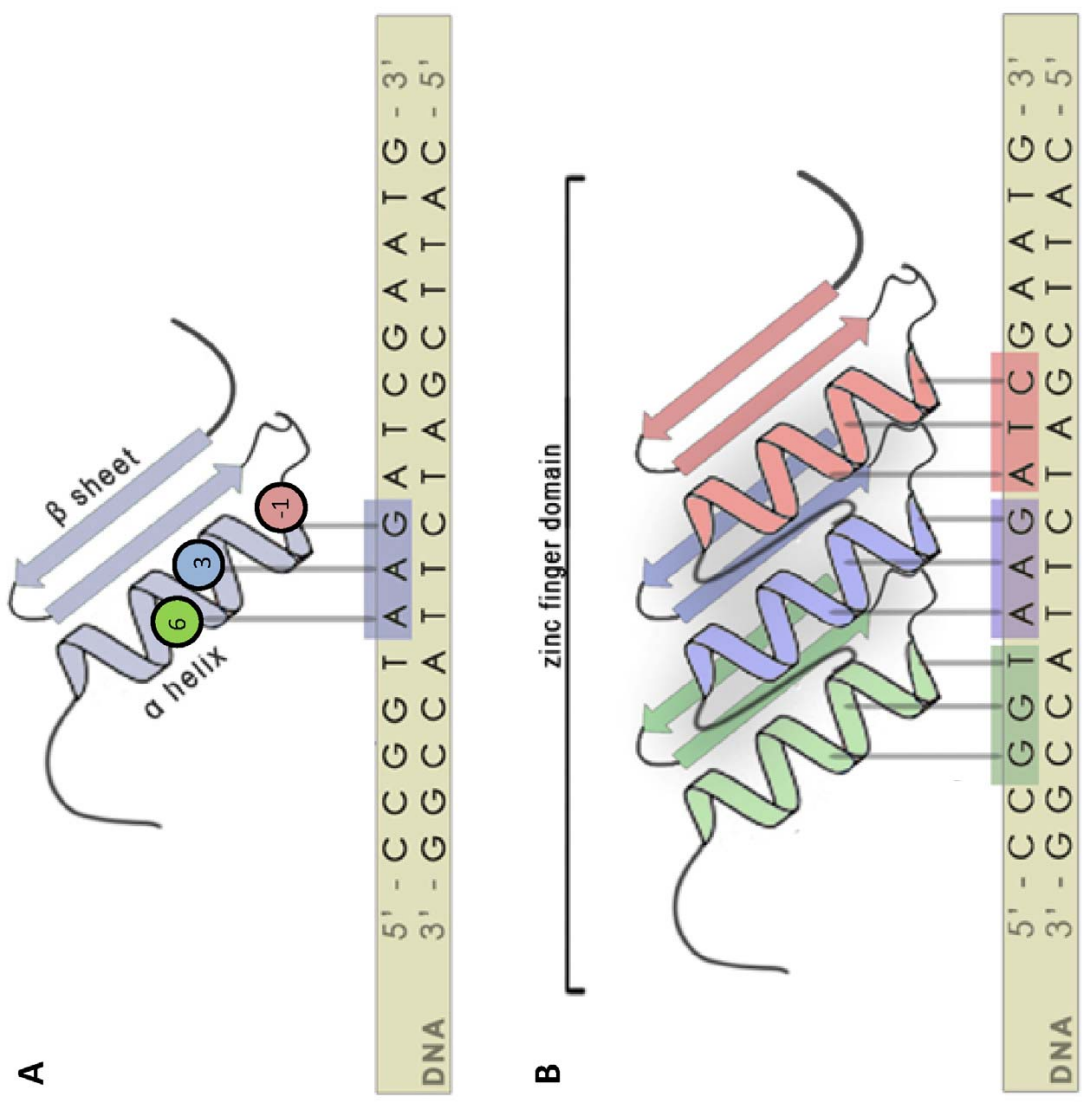
A model of Cys_2_His_2_ zinc finger binding. In A, one zinc finger is depicted with its ββα structure, where amino acid residues at positions −1, 3, and 6 in relation to the start of the α helix bind to DNA. In B, multiple zinc fingers are displayed making tandem contacts with DNA. (This figure is adapted from http://2010.igem.org/Team:Slovenia with permission from Roman Jerala).

Cys_2_His_2_ zinc fingers are among the most common DNA-binding motifs found in eukaryotic transcription factors. These zinc finger proteins usually contain multiple “fingers”, all of which have a conserved ββα structure with amino acids in the α-helix contacting DNA in the major groove of the double helix [12] (**Figure 1**). Zinc finger proteins function chiefly in protein-DNA binding, but also may be involved in protein-RNA binding and protein-protein binding, making them key elements in transcriptional regulation and many other processes. While transcription factors have long been recognized for their required role in yeast α recombination hotspots [13], the discovery of *Prdm9* is the first implication of zinc finger proteins and their predicted binding sequence motifs as major determinants of recombination hotspot location and usage in multi-cellular organisms [14].

Yeast and mammals share many conserved meiotic proteins and processes. However, the Drosophila meiotic recombination process differs from yeast and mammals in several key components. First, homologous chromosome pairing and synapsis proceed normally in the absence of double strand breaks in Drosophila [15], [16], [17], [18], [19], [20]. Indeed, Drosophila males undergo meiosis without any homologous recombination, a phenomenon rarely seen in other animals [21], [22]. Furthermore, the synaptonemal complex, a proteinaceous structure that binds homologs together during meiotic prophase, is conserved in structure but has diverged in function in Drosophila. The Drosophila synaptonemal complex does not require SPO11 to form, and functions in both the initiation of recombination and the facilitation of the formation of DSBs. Finally, Drosophila lack some genes known to be crucial in recombination in other organisms, like *Msh4* and *Msh5*, and use a smaller subset of proteins in DNA repair [16], [23].With known differences in meiotic proteins and some apparent differences in the initiation of recombination, it is unclear if Drosophila would possess a recombination initiation process involving a protein like PRDM9.

In 2011, Lake *et al.* demonstrated that the Cys_2_His_2_ zinc finger protein *trade embargo* is required for meiotic DSB initiation in *Drosophila melanogaster* and suggested its essential role for processing DSBs into crossovers [24]. However, while *Prdm9* binds to discrete sites across the genome, *trade embargo* appears to bind the entire length of the chromatin, casting doubt on the similarity between the two proteins. Nonetheless, the discovery of *trade embargo*’s role in DSB initiation and resolution implicates a general role for zinc finger proteins in the distribution of recombination.

Here, we explore the importance of Cys_2_His_2_ zinc finger genes in the initiation of Drosophila recombination and whether the abundance of predicted binding sites of such genes may correlate with recombination variation within and among species. First, we attempt to identify a *Prdm9* homolog in Drosophila, and confirm that *Prdm9* is indeed not detectable in this lineage. We then attempt to characterize any other zinc finger proteins involved in recombination by analyzing associations between predicted DNA sequence motifs and our empirically derived broad- and fine-scale measures of recombination rate in *D. pseudoobscura* and its close relative. As a validation of our approach, we apply the same procedure to the *Prdm9-*predicted motif using comparably scaled measures of recombination rate in humans. Our results suggest that Drosophila possess a recombination initiation mechanism disparate from human *Prdm9*.

## Materials and Methods

### System and Datasets

The species *Drosophila pseudoobscura* was selected due to the availability of high resolution recombination data not yet available in *Drosophila melanogaster*. Additionally, the availability of recombination data in closely related species *D. miranda* facilitates inter-specific comparisons. *D. pseudoobscura* inhabits the western coast of North America and diverged from *D. melanogaster* approximately 55 mya [25]. The recombination datasets for *D. pseudoobscura* consist of two recombination maps from the Flagstaff population (collected Flagstaff, AZ 1997), for more information about the recombination maps, see McGaugh *et al.* (2012) [26]. The “superfine” recombination map consists of three ∼100 kb regions on chromosome 2 with markers spaced within these regions every 20 kb (219 crossovers observed). Briefly, the map was constructed by genotyping over 10,000 F2 backcross progeny by PCR at 19 markers. Their coordinates on chromosome 2 are: 6.003 Mb- 6.108 Mb (6 markers, 5 intervals, average interval 20.280 kb), 17.534 Mb - 17.660 Mb (7 markers, 6 intervals, average interval kb 20.878), 21.438 Mb −21.537 Mb (6 markers, 5 intervals, average interval 19.870 kb). The “broad-scale” recombination map spans the majority of chromosome 2 with markers approximately every 180 kb, resulting in 140 intervals (1344 crossovers observed) [26]. The broad-scale map was constructed by genotyping 1440 individual backcrossed flies for 384 line-specific SNP markers using the Illumina BeadArray platform [27] (Illumina, San Diego, California, United States).

For comparisons across species, a “broad-scale” *D. miranda* recombination map of chromosome 2 was used. The *D. miranda* broad-scale map was constructed using the same method as the *D. pseudoobscura* broad-scale map, and SNP markers were designed at comparable physical coordinates. For the between-species comparison, both the *D. pseudoobscura* and *D. miranda* recombination maps were condensed to comparable interval sizes, yielding 97 windows of about 320 kb [26]. Chromosome 2 is 30 Mb and makes up 23% of the physical genome.

DNA sequence for the strains corresponding to the recombination maps was also obtained from McGaugh *et al.* (2012) [26]. We obtained the amino acid sequence for Cys_2_His_2_ zinc finger proteins for *D. melanogaster* and *D. pseudoobscura* from FlyBase [28], [29], for *D. persimilis* from FlyMine [29], and for *D. miranda* from our own sequence data [26].

### Identification of a Prdm9 Homolog using BLAST

We used NCBI BLAST protein tools blastp and PSI-BLAST and the nucleotide tool blastn with default parameters, specifying the organism as Drosophila [30]. For the input query, we examined all genes and proteins annotated as *Prdm9*, selecting *Homo sapiens*, *Strongylocentrotus purpatus*, and *Mus musculus* PRDM9 proteins and *Prdm9* sequence for input queries.

### Motif Prediction

We used custom Perl and Unix scripts to extract the zinc finger domains from each *D. pseudoobscura* protein using the canonical Cys_2_His_2_ binding pattern CX(2–6)CX(11–13)HX(2–6)H. Each Cys_2_His_2_ protein contains a number of zinc fingers ranging from one to 21, with an average of five in *D. pseudoobscura*. We used a protein only if it had more than one zinc finger, obtaining binding sequences longer than 3 base pairs. This procedure resulted in an amino acid dataset of 186 unique *D. pseudoobscura* proteins (**Table S1**). For each zinc finger, we recorded the amino acid residues at positions −1, 3, and 6 in relation to the start of the alpha helix, which are responsible for predicting DNA binding specificity [31]. To generate the DNA sequence that these amino acids are predicted to bind to, we used two approaches.

To examine candidate proteins containing a SET domain, those identified in our BLAST searches, or ontologically identified as functioning in meiosis, we used the rigorous approach of Baudat *et al.* (2010) to generate the sequence motif for *Prdm9*. Briefly, we used the Zinc Finger Consortium database to obtain a matrix of binding residues, positions, and empirically determined binding sequence [32], then input this data into WebLogo to generate the sequence motif [33]. To take into account that not all zinc fingers may be important in binding, we used a 3 letter sliding window for the DNA sequence motifs, looking at the whole motif and all possible contiguous 9 bp motifs from the whole motif. This approach was applied to zinc finger proteins GA18168 (*trade embargo*), GA23469 (*Blimp-*1), GA25755 (*hamlet*), GA26409 (CG9817), GA25849 (*crooked legs*), GA26228 (CG5245), GA26117, GA21024 (*combgap*), GA21437 (*teflon*), and GA17308 (*grauzone*) (**Table 1**).

**Table 1:** PRDM9 candidate proteins.

Gene name (*D. melanogaster* homolog)	Sequence Motif	Protein Domains or notes
GA23469 (*Blimp-1*)	TGA[TG]ANGGA[GT]AA	SET domain, 4 zinc fingers
GA25755 (*hamlet*)	GAAGATGAGGAANNTGN[CT]NNC	SET domain, 7 zinc fingers
GA26409 (*CG9817*)	NCTTA[AT]NGAGAN[TG]N[TC]	SET domain, 5 zinc fingers
GA25849 (*crooked legs*)	GAC[TG]GNNA[TC]GGGGGGGGGGGGGGGGGGGGGGGGGGGGGG	15 zinc fingers
GA26228 (*CG5245*)	[GT][TC]CGNGGGGTNCTNC	6 zinc fingers
GA26117	A[TG][CT]GNNTC[CT]GC[CT][GT][GC]ATNNTNCAN[TC][TG]GANG[TC]GA[TC]	11 zinc fingers
GA21024 (*combgap*)	NN[CT][TG][TC]NN[CT]TNACGNGNGA[TG]G[TC][TG]G[TC][TG]N[TC][TG]G[TC]	10 zinc fingers
GA18168 (*trade embargo*)	TGGNANGCCG[CG]ACNT	5 zinc fingers; meiotic protein
GA21437 (*teflon*)	GNGGNNG[TC][TC]	3 zinc fingers; meiotic protein
GA17308 (*grauzone*)	NANGNN[TG][TC]NNACG[TC]C[TG][TC]GN[TC]NGNC	8 zinc fingers; meiotic protein

Included in this table are all zinc finger proteins identified as PRDM9 candidate proteins. These proteins were chosen through BLAST results, presence of a SET domain, and/or function in meiosis. Gene name is given as *D. pseudoobscura* with *D. melanogaster* homolog in parentheses. Sequence motifs are listed as the full predicted motif for a given gene. An “N” indicates that there was not enough information to accurately predict a nucleotide at that position in the motif. Square brackets ([]) indicate that any nucleotide enclosed within them is acceptable at that position in the motif.

For all other zinc finger proteins, we used the more scalable program enoLOGOS, with default parameters [34]. The input for this program simply requires the amino acid contact residues for each zinc finger. The output is a normalized sequence logo of nucleotides, with the information content of each nucleotide position measured in bits (ranging from zero to two). Again, we used a 3 letter sliding window for the DNA sequence motifs, looking at the whole motif and all possible contiguous 9 bp motifs from the whole motif.

### Motif Occurrence

DNA sequence for *D. pseudoobscura* Flagstaff was split into intervals based upon the windows in which recombination was surveyed (see “Systems and Datasets” section above, and McGaugh et al., 2012 [26] for more information). This resulted in 140 windows of average size 180 kb for the *D. pseudoobscura* Flagstaff broad-scale dataset and 16 windows of average size 20 kb for the *D. pseudoobscura* Flagstaff superfine-scale dataset. To identify the frequency of occurrence of all *D. pseudoobscura* zinc finger motifs, we used the EMBOSS command “dreg” [35]. The command “dreg” searches one or more sequences with the supplied regular expression and writes a report file with the matches. The frequency of motifs in a given interval for forward and reverse strands was combined and corrected for interval size, then regressed with recombination rate using custom Perl and R scripts. p-values were adjusted for multiple comparisons using a sequential Bonferroni correction [36]. For proteins that were significantly associated with recombination after correction for multiple comparisons, we ran a multiple regression accounting for total GC content (JMP Version 9.0. SAS Institute Inc., Cary, NC). Using other measures of GC content (e.g., non-coding only) did not alter results.

### Amino Acid Differences between Species

To identify changes in Cys_2_His_2_ zinc fingers that alter DNA binding, we compared number of fingers and amino acids at positions −1, 3, and 6 for each protein in *D. melanogaster*, *D. miranda*, and *D. persimilis* to *D. pseudoobscura* using a custom Perl script. After identifying proteins that had differences in their zinc fingers between *D. pseudoobscura* and *D. miranda*, we followed the protocol outlined in the Motif Occurrence section above, but using the condensed *D. miranda* recombination data and sequence with this subset of proteins. The frequency of predicted motifs for this subset of *D. miranda* zinc finger proteins was identified using *D. miranda* recombination and sequence and *D. pseudoobscura* recombination and sequence, and then the correlation coefficients compared. The same was done for predicted motifs for the same subset of *D. pseudoobscura* zinc finger proteins.

### Candidate Motif Analysis

To identify any overrepresented sequence motifs not *a priori* associated with zinc finger binding, we used the EMBOSS command “wordcount,” which counts and extracts all possible unique sequence words of a specified size in one or more DNA sequences. This analysis was done using a word size of six with the superfine-scale and broad-scale recombination datasets. To identify associations with recombination rate, the forward and reverse complement motif counts were combined and the motifs with the highest frequency difference between the highest and lowest recombination intervals were noted. Following Cirulli *et al.* (2007), the two windows (six windows for the broad-scale) used were excluded and the frequency of the subset of motifs was regressed using the remaining windows. Results were corrected for multiple comparisons using a sequential Bonferroni correction. Additionally, we analyzed the human motif CCNCCNTNNCCNC [4], [37] and the *D. melanogaster* motif GTGGAAA [38] using the approach described above in the Motif Occurrence section above.

### Human Comparison

We obtained human recombination data from Kong *et al.* (2002) [39] and genome sequence from a Finnish population, a part of the 1000 Genomes Project [40]. As above, the sequence was partitioned into intervals of known recombination across human chromosome 1 (used because of its large size). Using the same EMBOSS script “dreg,” motif frequency of the 13-mer degenerate motif CCNCCNTNNCCNC [3], [4] was tallied and a regression looking at motif frequency corrected for interval size and recombination rate was performed. Recombination intervals used for the regression were restricted to the same number of windows and similar recombination range of our *D. pseudoobscura* recombination data (Number of intervals = 140 for both datasets; *D. pseudoobscura* cM range: 0.079–3.97, mean: 0.765, median: 0.487; Human cM range: 0.142–3.11, mean: 0.693, median: 0.505).

## Results

### Prdm9 Homology

Oliver *et al.* (2009) suggested that, although *Prdm9* is essential for fertility in mice, it appears to be absent in *Drosophila melanogaster* and its function in meiosis may be lineage or even species-specific. Previous studies support this conclusion, with the expansion of the PRDM gene family postdating the split between Drosophila and Echinoderms and Chordates, and about 61% of genes having identifiable homologs between Drosophila and human [41], [42], [43]. Drosophila are recognized to have only three members of this gene family: PRDM1 (Blimp-1), PRDM5 (CG9817), and hamlet [42]. To confirm that PRDM9 is indeed not identifiable in the Drosophila genus, we BLASTed *Prdm9* and PRDM9 against Drosophila species. Using human, sea urchin, and mouse protein input queries with the BLAST tools blastp and PSI-BLAST, we identified the genes *GA26117*, *CG5245*, *crooked legs*, *meics*, *combgap*, *CG9817*, *Blimp-1*, *hamlet*, and *trithorax-related*. All Drosophila proteins identified using BLAST contained zinc finger domains, and *CG9817*, *Blimp-1*, and *trithorax-related* contained SET domains. The maximum amino acid sequence identity ranged between 49% and 38% and the part of the query sequence that was covered ranged between 98% and 73%. Nucleotide input queries using blastn yielded results with a maximum identity between 84% to 97%, but the query only covered between 1% to 13% of the nucleotide sequences of the surveyed genes. These BLAST results, combined with previous data, suggest there is not a *Prdm9* homolog detectable in Drosophila. However, genes identified in this manner, which are proteins that possess SET domains and/or zinc fingers, are candidates that may function similarly to PRDM9.

### Candidate Protein Analysis

To identify if any proteins function in Drosophila recombination in a similar manner to PRDM9 in humans, we selected a subset of *D. pseudoobscura* Cys_2_His_2_ zinc finger proteins as candidates. Proteins were selected as candidates if they were: identified using BLAST (above), possessed an annotated SET domain, and/or were involved in meiotic recombination in Drosophila [29] (**Table 1**).

Cys_2_His_2_ zinc finger DNA binding residues are determined by amino acids at positions −1, 3, and 6 in relation to the start of the alpha helix [31]. We recorded the amino acid binding residues for all BLAST, SET domain, and meiotic protein candidates, and obtained the predicted nucleotide targets using the same approach taken to identify the binding preferences of human PRDM9 [3]. Once a consensus motif was established for each protein, a 3 base pair sliding window of 9 base pairs was used for each motif, as the binding length for a protein with N fingers is 3N, but not all zinc fingers may be used in binding. As zinc fingers bind in sequential tandem, this approach should capture all possible binding configurations. Motif occurrence was then analyzed using two *D. pseudoobscura* sequence and recombination datasets: a “superfine-scale” recombination map and a “broad-scale” recombination map. The superfine-scale dataset surveys recombination in 16 intervals of approximately 20 kb in size over 3 Mb of chromosome 2. This dataset was constructed using over 10,000 individuals and contains 219 observed crossover events. The broad-scale dataset estimates recombination in 140 intervals of approximately 180 kb in size across all of chromosome 2 (30 Mb) [26]. This dataset was constructed by genotyping approximately 1400 individuals at 384 markers across the genome and captured 1344 crossover events.

Motif frequency was regressed with recombination rate, and after correcting for multiple comparisons, no motifs were significantly associated with recombination at the superfine-scale, and three sequence motifs were significantly associated with recombination at the broad-scale. A multiple regression correcting for total GC content did not alter the results, so the numbers reported below reflect a simple linear regression (though the statistics for the multiple regression are reported in Table 2 in parentheses). These proteins are *crooked legs* (p = 0.003, r = 0.331), which functions in lateral inhibition, cell adhesion, and negative regulation of transcription; GA26117 (p = 0.019, r = −0.296), of unknown biological function; and *combgap* (p = 0.0253, r = −0.290), which functions in imaginal disc-derived wing morphogenesis. These results, while significant, are not particularly compelling due to the high repeat content in the *crooked legs* motif, and the highly degenerate nature of the other two motifs (**Table 2**). Of note, *trade embargo* was not significantly associated with recombination at either scale, providing support that it may not bind to discrete foci [24].

**Table 2:** Zinc finger proteins with predicted sequence motifs significantly associated with recombination.

Gene (*D. melanogaster* homolog)	Predicted Sequence Motif	Association between motif and recombination at the superfine-scale:p, r (GC content corrected p, r)	Association between motif and recombination at the broad-scale: p, r (GC content corrected p, r)
GA25849 (crooked legs)	GGGGGGGGG	0.9106, −0.0306 (0.8514, 0.1564)	0.0034, 0.3307 (0.0003, 0.3321)
GA26117	TNNTNCAN[TC]	0.3510, −0.2497 (0.3050, 0.4086)	0.01923, −0.2963 (0.0015, 0.3013)
	AN[TC][TG]GANG[TC]	0.9159, 0.0287 (0.5814, 0.2829)	0.0272, −0.2882 (0.0012, 0.3050)
GA21024 (combgap)	[TG][TC]NN[CT]TNAC	0.3241, 0.2635 (0.5318, 0.3043)	0.0253, −0.2902 (0.0017, 0.2979)
GA15299 (CG2202)	N[GA]GGGGGGG	0.8288, −0.0588 (0.8399, 0.1627)	<0.0001, 0.4723 (<0.0001, 0.4702)
GA21173 (su(Hw))	[CA][CT]TNAG[GC]T	0.2679, 0.2946 (0.8471, 0.1588)	<0.0001,−0.4444 (<0.0001, 0.4488)
GA12131 (zfh1)	GTTANNNTN	0.7078, 0.1017 (0.8518, 0.1561)	0.0050,−0.3676 (<0.0001, 0.3678)
GA22134 (CG9932)	NNTANN[GC][TC]N	0.4855, −0.1881 (0.5491, 0.2968)	0.0083,−0.3592 (<0.0001, 0.3621)
GA14502 (Oaz)	[GC]TTANNGNN	0.1056, −0.4197 (0.3145, 0.4037)	0.0166,−0.3474 (0.0001, 0.3535)
	TNTT[CA][GA]G	0.5369, −0.1668 (0.7461, 0.2099)	0.0234,−0.3413 (0.0002, 0.3413)
GA20521 (CG7691)	NACNTN	0.1618, −0.3672 (0.5137, 0.3121)	0.0219,−0.3424 (0.0001, 0.3481)
GA11205 (charlatan)	NNTN[TG]GG[AT]C	0.4588, −0.1995 (0.8200, 0.1734)	0.0328, −0.3351 (0.0002, 0.3401)
GA11270 (CG11902)	[CA]ATN[TG]G[GC]A[CT]	0.1401, 0.3857 (0.7821, 0.1926)	0.0389,−0.332 (0.0003, 0.3374)
GA15842 (CG30431)	NNTATT[GC]NG	0.9141, 0.0293 (0.7495, 0.2083)	0.042,−0.3305 (0.0003, 0.3352)

Gene name is given as *D. pseudoobscura* with *D. melanogaster* homolog in parentheses. The motif is a partial or whole motif significantly associated with recombination at the broad scale (no motifs were significantly associated with recombination at the superfine-scale). An “N” indicates that there was not enough information to accurately predict a nucleotide at that position in the motif, so any nucleotide is acceptable at that position. Square brackets ([]) in the motif column indicate either letter enclosed is acceptable at that position. The “broad-scale” column indicates the p-value (corrected for multiple comparisons) and correlation coefficient (r) for the broad-scale recombination dataset. Although these motifs were not significantly associated with recombination rate, the p-value and correlation coefficient (r) for the superfine-scale recombination dataset are included for reference. The p-values and correlation coefficients (r) from multiple regressions correcting for total GC content are included in parentheses.

### A Comprehensive Search for a Zinc Finger Binding Sequence Motif

Due to the negative results obtained in the candidate protein analysis, we expanded our analysis to determine if *any D. pseudoobscura* Cys_2_His_2_ zinc finger proteins are associated with recombination. A more stream-lined approach was taken to identify predicted sequence motifs in the comprehensive search than in the candidate protein analysis. The amino acid binding residues of all *D. pseudoobscura* zinc finger proteins were recorded, and predicted nucleotide targets were generated using the program enoLOGOS [34]. Sequence motifs for 186 *D. pseudoobscura* proteins were identified (**Table S1**). Again, a 3 base pair sliding window of 9 base pairs was used for each motif, and motif occurrence was determined and analyzed using both the superfine- and broad-scale *D. pseudoobscura* sequence and recombination datasets [26]. The superfine-scale recombination dataset was analyzed using all nucleotide sequence *and* strictly intergenic sequence, while the broad-scale dataset was analyzed using all nucleotide sequence. Intergenic sequence was used for the superfine-scale recombination dataset because recombination is known to commonly initiate in intergenic regions in yeast [13], [44].

After correcting for multiple comparisons, no Cys_2_His_2_ zinc finger protein analyzed at the superfine-scale was significant. This is complicated by the fact that there are hundreds of comparisons and only 16 windows of known recombination, so power to detect significance is low. At the broad-scale, ten proteins were significantly associated with recombination after correction for multiple comparisons (**Table 2**). Again, a multiple regression correcting for total GC content did not alter the results. Only one protein, GA15299 (CG2202), was positively associated with recombination. The remaining nine proteins were negatively associated with recombination, which may be expected for a protein like suppressor of Hairy wing (*su(Hw)*) that functions in the negative regulation of transcription and negative regulation of chromatin silencing, but contradicts the expectation from PRDM9. Furthermore, there was no overlap in zinc finger motifs between the superfine-scale and broad-scale analyses, which casts doubt on the detected associations.

### Differences in Motif Occurrences do not Account for Changes in Recombination Landscapes between Closely Related Species

PRDM9 is known to be undergoing rapid positive selection, changing both the number of zinc fingers present and the DNA-binding amino acid residues at positions −1, 3, and 6 [45], [46]. To determine Cys_2_His_2_ zinc finger proteins changing rapidly across the Drosophila lineage, we compared number of zinc fingers present and number of changes in binding residues for each Cys_2_His_2_ protein in *D. melanogaster* (55 mya), *D. miranda* (3 mya), and *D. persimilis* (0.5–1 mya) to *D. pseudoobscura*.

**Figure 2:**
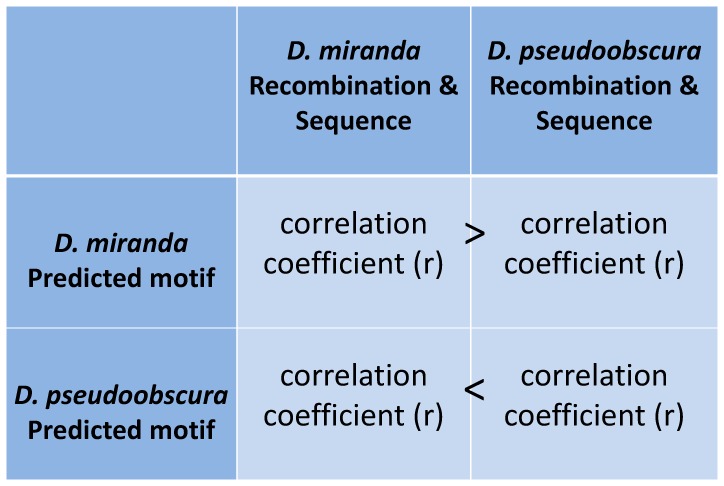
Predictions to test if changes in DNA binding motifs between species account for variation in recombination rate between species. This figure depicts predictions testing if variation in the zinc finger binding sites between *D. miranda* and *D. pseudoobscura* is accountable for variation in recombination rates between species. After detecting which zinc finger proteins differed between these two species, we generated new predicted motifs for this subset of *D. miranda* zinc fingers. We then found the frequency of the motif and any associations with recombination using *D. miranda* sequence and recombination. We then took these *D. miranda* predicted motifs and repeated using *D. pseudoobscura* sequence and data. If changes in the zinc finger proteins were accountable for the variation in recombination rate between species, one expects to see a stronger correlation between *D. miranda* predicted motifs with *D. miranda* sequence and recombination data than *D. miranda* predicted motifs with *D. pseudoobscura* sequence and recombination data. Similarly with *D. pseudoobscura*, one expects to see a stronger correlation between *D. pseudoobscura* predicted motifs with *D. pseudoobscura* sequence and recombination data than *D. pseudoobscura* predicted motifs with *D. miranda* sequence and recombination data. If these predictions are not met, one can conclude that changes in the DNA binding motifs between these two species do not account for changes in recombination rate.

Between *D. pseudoobscura* and *D. miranda*, a large majority of Cys_2_His_2_ zinc finger protein binding residues are conserved. To identify any changes in recombination rate associated with change in binding of Cys_2_His_2_ zinc finger proteins, we generated new sequence motif predictions for proteins with mismatches between *D. miranda* and *D. pseudoobscura*. The recombination map in *D. miranda* was constructed using markers with the same physical coordinates as the *D. pseudoobscura* broad-scale map, making the two maps directly comparable. While recombination rates from the two maps are correlated, the *D. miranda* chromosome 2 recombination rate is approximately 1.3 times higher than *D. pseudoobscura* (rare events logistic regression, z-value −4.4974 p<0.001) [26]. Utilizing these *D. miranda* and *D. pseudoobscura* broad-scale recombination maps, we then compared the association between *D. miranda* binding motifs and *D. miranda* recombination to *D. miranda* binding motifs and *D. pseudoobscura* recombination and then repeated with comparing *D. pseudoobscura* motifs to *D. pseudoobscura* and *D. miranda* recombination. If a protein is involved in recombination, we expect to see a stronger correlation between binding motif and recombination within species than between species (**Figure 2**).

Comparing all associations with an un-corrected p-value <0.05, no protein was consistently more strongly correlated with recombination within species than between species. Therefore, we conclude that no differences associated with zinc finger binding are responsible for recombination rate changes between these species of Drosophila.

### Sequence Motifs

To identify overrepresented sequence motifs without an identified association with zinc fingers, all possible 6 base pair motifs were analyzed for frequency using the superfine- and broad-scale recombination datasets. The 6 bp length was selected because motifs of greater length were typically composed of repeating motifs encompassed in the 6 bp length motif. Ten motifs with the greatest frequency difference between regions of high and low recombination intervals were selected and regressed with recombination rate. At the broad-scale, the motifs AATAAA (p = 0.0397, r = −0.178) and CTGCTG (p = 0.0539, r = −0.1669) were weakly, negatively associated with recombination, and the motifs CTCTCT (p = 0.0115, r = 0.0115) and TCTCTC (p = 0.0126, r = 0.2149) were weakly, positively associated with recombination. At the superfine-scale, the motifs AAATTT (p = 0.0717, r = 0.4954) and ACAAAT (p = 0.0594, r = 0.5151) were weakly, positively associated with recombination.

Previous studies in Drosophila found associations between local recombination rates and the human *Prdm9* motif CCNCCNTNNCCNC [37], and the *D. melanogaster* motif GTGGAAA [38]. In this study, neither of these previously described motifs were significantly associated with recombination rate variation in *D. pseudoobscura* at either scale, although this lack of association is not unexpected as these motif associations were detected previously in different species.

### A Validation of Our Approach using Human Recombination Data

To assess if one can detect an association between a sequence motif and recombination rate using relatively coarse recombination rate estimates, we utilized recombination data from an Icelandic population that empirically surveyed genome wide recombination in 869 individuals (average window size: 650 kb). We restricted the dataset to a subset of chromosome one with a comparable recombination range to *D. pseudoobscura* (see Materials & Methods for details). A regression between the frequency of the human *Prdm9* motif CCNCCNTNNCCNC and recombination rate was positive and statistically significant (p = 0.0004, r = 0.3), thus demonstrating sequence motif signals can be detected in humans with broad-scale recombination data comparable to that used in the Drosophila studies.

## Discussion

Our attempts to identify a PRDM9-like protein involved in meiotic recombination initiation in Drosophila yielded negative results. Generating predicted nucleotide sequence motifs from Cys_2_His_2_ zinc finger proteins and regressing their frequency with estimated recombination in *D. pseudoobscura* produced a handful of recombination associated sequence motifs, but the biological relevance of these associations remains uncertain. Furthermore, changes in the binding motifs between species do not appear to account for variation in the recombination landscape. Our results could be complicated by the approach taken, or alternatively, we suggest these findings could be explained by the existence of a different recombination initiation system in Drosophila.

### Approach

Our results could be due in part to the scale at which recombination was estimated in *D. pseudoobscura*. While the superfine- (20 kb) and broad-scale (18 0 kb) recombination datasets used represent one of the most comprehensive recombination maps outside human, mouse, and yeast, the datasets might still lack the resolution needed to determine sequence motifs associated with recombination. Successful work with sequence motifs in yeast and human recombination has been analyzed at a scale <1–2 kb [4], [44], [47], [48], [49], although we were able to detect a strong association here between the frequency of the human *Prdm9* motif and human recombination rate using broader (∼650 kb intervals), comparable in recombination to what we studied, thereby validating our approach. Furthermore, recombination associated motifs have been identified at scales ranging from 220 kb to 5 Mb in other organisms [37], [50], [51], [52], [53].

Additionally, there is an inherent limitation in one of the bioinformatic approaches utilized here, in the ability of currently developed programs to accurately identify DNA binding motifs of zinc fingers. While algorithms have improved over the years, it is impossible to be certain that identified motifs are “correct.” Hence some motifs predicted in this manner could be biologically irrelevant. We attempted to address this problem in two ways. First, for BLAST, SET domain, and meiotic candidate zinc finger proteins, we followed a proven motif prediction protocol: the methods utilized to identify the binding nucleotides for PRDM9 [3].Because of its success in determining the PRDM9 associated sequence motif, we can be somewhat more confident in concluding that our motif predictions for these proteins are correct, and therefore, that no Drosophila candidate proteins we tested are associated with recombination. Second, we used an unbiased approach to identify all motifs of six base pairs in length and to test their association with recombination, although even this approach is imperfect since it is not possible to search for degenerate motifs of all possible lengths. Despite these accommodations, it remains possible that Cys_2_His_2_ zinc finger protein associated sequence motifs do play a role in Drosophila meiotic recombination, but that it is beyond the scope of the technology to detect them at this point in time.

### A Different Recombination Initiation System in Drosophila?

Alternatively, it is possible and likely that other factors play a major role in the determination of recombination in Drosophila. Historically, it has been thought that Drosophila do not have the 1–2 kb hotspots characteristic of yeast, humans, and mice [54], [55], [56], [57]. This is supported by the lack of apparent hotspots of intragenic recombination in *rosy* [58], [59], [60] and in *white-echinus* [61], and the lack of linkage disequilibrium among nearby nucleotides as compared to humans [56], [57], [62]. The human *Prdm9* recombination initiation model is based on the specific targeting and binding of the PRDM9 protein to a sequence motif, enriched in recombination hotspots (although this model may need some refining, see below). If Drosophila lack such recombination hotspots, this evidence supports Drosophila lacking a recombination initiation system that functions in a sequence specific binding function like *Prdm9* in humans, although obviously cannot rule out a sequence binding independent function.

Additionally, Drosophila recombination is known to differ from other organisms [15], [16], [17], [18], [19], [20]. First, homologous chromosome pairing and synapsis proceed normally in the absence of double strand breaks. Second, the synaptonemal complex does not require SPO11 to form and functions in the initiation of recombination and the facilitation of the formation of DSBs. Third, Drosophila are missing some genes known to be crucial in recombination in other organisms [16]. With these known differences in meiotic proteins, and apparent differences in the initiation of recombination, this evidence is supportive of Drosophila possessing a different recombination initiation process than humans.

Furthermore, *Prdm9* is missing or altered in many organisms [42], [45], [46], necessitating the existence of alternative recombination initiation systems. The PRDM family is absent in plants and fungi, and is quite small in other taxa, with only two genes in nematodes and three genes in arthropods. While PRDM9 functions in meiotic recombination in both mouse and human, it seems as if this function is lineage specific. *Prdm9* is non-functional in canines [63], [64], and is missing all zinc fingers in the marsupial *Monodelphis domestica* [46], so even amongst mammals, recombination initiation may vary.

Finally, the PRDM9 story is made more complex by a general lack of understanding of the *in vivo* function of PRDM9 (although see [10]). In humans, although the PRDM9 motif is only detected in a proportion of hotspots, data suggest that PRDM9 influences hotspot activity even at hotspots in which the motif is absent [6], [14], [65]. While there is *in vitro* evidence that the zinc fingers of PRDM9 do bind to the motif, this suggests PRDM9 interacts with hotspots genome wide in a more complex and subtle way than first expected. Furthermore, the predominant human sequence motif is neither necessary nor sufficient to drive hotspot activity in humans, with the motif represented approximately 290,000 times in the genome and only about 50,000 detectable hotspots [14]. In chimpanzees, there is extensive variation in the PRDM9 protein, and little evidence of any sequence motifs enriched in hotspots [66]. Researchers suggest the most plausible explanation for this observation is that PRDM9 may still play the same roles in chimpanzee as it does in mouse and human, but the PRDM9 alleles may bind to a much greater variety of sequence motifs than seen in human. This implies that other factors, like chromatin state, play a more dominant role in the hotspot localization. Taken in this context, our data could suggest that there is a PRDM9-like protein in Drosophila, but it either binds a wide repertoire of sequence motifs, or functions in a sequence-specific-binding independent manner.

Regardless of the model, given recent observations that PRDM9 influences more human recombination hotspots than previously thought, and possibly all hotspots [14], it is quite remarkable that a single protein rapidly evolved to play such a critical role in recombination in the human lineage. Recombination is an essential mechanistic and evolutionary process, so *Prdm9* poses an intriguing step in the evolution of meiosis. However, *Prdm9* appears to be only a piece of the puzzle when looking at recombination across taxa. Evidence from Drosophila and other organisms suggests that *Prdm9* is not the quintessential element defining meiotic recombination; instead, there remain many mysteries to explore.

## Supporting Information

Table S1 ***D. pseudoobscura*** **predicted zinc finger motifs.** The gene name is listed in column one. In column two, each row represents a zinc finger within that particular protein. The amino acids at positions −1, 3, and 6 are indicated with their corresponding predicted nucleotide binding motif (column 3). The whole motif is listed in column four in the orientation it would be found in the sequence.
(DOCX)
