# Supplementary material for: Zinc Finger Binding Motifs Do Not Explain Recombination Rate Variation within or between Species of Drosophila
Source: PLoS One. 2012 Sep 18;7(9):e45055. doi: 10.1371/journal.pone.0045055 (PMC3445564; doi:10.1371/journal.pone.0045055)
Supplement: Table S1 — D. pseudoobscura predicted zinc finger motifs. The gene name is listed in column one. In column two, each row represents a zinc finger within that particular protein. The amino acids at positions −1, 3, and 6 are indicated with their corresponding predicted nucleotide binding motif (column 3). The whole motif is listed in column four in the orientation it would be found in the sequence. (DOCX) [file pone.0045055.s001.docx]

| **Name** | **Individual zinc finger amino acids (-1,3,6)** | **Corresponding Predicted Motif** | **Predicted motif (whole)** |
| --- | --- | --- | --- |
| **GA11485** | NLD | N[TC]T | N[TC]T |
|  |  |  |  |
| **GA21038** | GEV | NNT | NATNNG[GT]TTGNNGTTA[CT]TG[CT]TGTANNT |
|  | QSL | GTA |  |
|  | SVR | G[CT]T |  |
|  | TTQ | A[CT]T |  |
|  | GAR | GTT |  |
|  | TGR | GNN |  |
|  | SAH | [GT]TT |  |
|  | REE | NNG |  |
|  | SNA | NAT |  |
|  |  |  |  |
| **GA18799** | NKE | NTT | NTTNTT |
|  | LAA | NTT |  |
|  |  |  |  |
| **GA11636** | SSN | NTT | NNTGNNNNCANNNTT |
|  | NEY | ANN |  |
|  | DQN | NNC |  |
|  | TGR | GNN |  |
|  | MQT | NNT |  |
|  |  |  |  |
| **GA17308** | DGE | NNC | NATNTNNTTAAC[TG][CT]CGTGN[CT]TNNC |
|  | NVA | N[CT]T |  |
|  | RLL | GTG |  |
|  | DVK | [TG][CT]C |  |
|  | DNQ | AAC |  |
|  | NAG | NTT |  |
|  | KSE | NTN |  |
|  | SNS | NAT |  |
|  |  |  |  |
| **GA11481** | TEN | NNT | GTTGCTNGNGATGTNGATGATGTCNTTGATGTTNNT |
|  | SSR | GTT |  |
|  | TNR | GAT |  |
|  | NAG | NTT |  |
|  | DAR | GTC |  |
|  | TNR | GAT |  |
|  | LNR | GAT |  |
|  | WSR | GTN |  |
|  | TNR | GAT |  |
|  | EHE | NGN |  |
|  | SDR | GCT |  |
|  | LSR | GTT |  |
|  |  |  |  |
| **GA10742** | NLQ | ATT | TGGGCTGATTTGTTGATT |
|  | YAK | TTG |  |
|  | YSK | TTG |  |
|  | SNL | GAT |  |
|  | GDR | GCT |  |
|  | RYK | TGG |  |
|  |  |  |  |
| **GA10243** | QGN | NNA | ANTTATGTTNTTNNA |
|  | GMM | NTT |  |
|  | SIR | GTT |  |
|  | SNW | TAT |  |
|  | SGQ | ANT |  |
|  |  |  |  |
| **GA17730** | TAQ | ATT | [TG]TC[TG]CT[CA]AGATT |
|  | RNI | [CA]AG |  |
|  | NDK | [TG]CT |  |
|  | DSK | [GT]TC |  |
|  |  |  |  |
| **GA17731** | KNI | [CA]AN | NTTN[CT]ANGG[CA]AN |
|  | RHD | NGG |  |
|  | QTV | N[CT]A |  |
|  | QNS | NTT |  |
|  |  |  |  |
| **GA19687** | SAD | NTT | NTT |
|  |  |  |  |
| **GA16696** | RTT | N[CT]G | GCGNAA[TG]CAN[CT]G |
|  | QDK | [TG]CA |  |
|  | QNT | NAA |  |
|  | RDR | GCG |  |
|  |  |  |  |
| **GA17373** | SHS | NGT | [TG]NTNGT |
|  | TQK | [TG]NT |  |
|  |  |  |  |
| **GA20696** | SGA | NNT | NAAG[GA]TTTN[GT]TGNNTNNT |
|  | SGG | NNT |  |
|  | RRH | [GT]TG |  |
|  | HAK | TTN |  |
|  | THR | G[GA]T |  |
|  | QNA | NAA |  |
|  |  |  |  |
| **GA14741** | SQK | TNT | [GT]NTN[CT]N[GT][CT]T[GA]TAGAG[GC]GCTNT |
|  | DYL | [GC]GC |  |
|  | RNR | GAG |  |
|  | QLN | [GA]TA |  |
|  | STH | [GT][CT]T |  |
|  | ETM | N[CT]N |  |
|  | NEH | [GT]NT |  |
|  |  |  |  |
| **GA18132** | QTN | [GA][CT]A | [GA][CT]A[GA][CT]A[GA][CT]A[GA][CT]A[GA][CT]A[GA][CT]A[GA][CT]A[GA][CT]A[GA][CT]A |
|  | QTN | [GA][CT]A |  |
|  | QTN | [GA][CT]A |  |
|  | QTN | [GA][CT]A |  |
|  | QTN | [GA][CT]A |  |
|  | QTN | [GA][CT]A |  |
|  | QTN | [GA][CT]A |  |
|  | QTN | [GA][CT]A |  |
|  | QTN | [GA][CT]A |  |
|  |  |  |  |
| **GA14497** | SDL | [GC]CT | GAA[TG]AAGAAGAAGAT[GC]CT |
|  | NNR | GAT |  |
|  | QNR | GAA |  |
|  | QNR | GAA |  |
|  | QNK | [TG]AA |  |
|  | QNR | GAA |  |
|  |  |  |  |
| **GA17013** | FFT | NAG | NAG |
|  |  |  |  |
| **GA18514** | TKL | [GC]TT | [TG]TTNGN[TG]CT[TG]G[TC]NNT[GC]TT |
|  | TET | NNT |  |
|  | LHK | [TG]G[TC] |  |
|  | NDK | [TG]CT |  |
|  | KYV | NGN |  |
|  | SLK | [TG]TT |  |
|  |  |  |  |
| **GA13192** | KIS | NTN | NNT[GA]TNGNCNNANTN |
|  | QEA | NNA |  |
|  | DER | GNC |  |
|  | HIN | [GA]TN |  |
|  | LQA | NNT |  |
|  |  |  |  |
| **GA10207** | KRE | NTN | TTA[GT]TN[GC]AT[TG]T[CT]NTN[GA]TTNNTNTNTT[CT]CTG[CA][AT]TNTN |
|  | TGI | [CA][AT]T |  |
|  | RLF | CTG |  |
|  | CAK | TT[CT] |  |
|  | IAD | NTN |  |
|  | TEA | NNT |  |
|  | NAN | [GA]TT |  |
|  | KAV | NTN |  |
|  | CSK | [TG]T[CT] |  |
|  | NNL | [GC]AT |  |
|  | KIH | [GT]TN |  |
|  | QAK | TTA |  |
|  |  |  |  |
| **GA19045-PC** | HKQ | ATN | TGGGNGNGNATN |
|  | KHA | NGN |  |
|  | RER | GNG |  |
|  | RHK | TGG |  |
|  |  |  |  |
| **GA15902** | KHS | N[GA]N | TGGGCGTGGGNGN[GA]N |
|  | RER | GNG |  |
|  | RHK | TGG |  |
|  | RDR | GCG |  |
|  | RHK | TGG |  |
|  |  |  |  |
| **GA11784** | GAE | NTT | [GC]G[TC][TG]NTNTT |
|  | SEK | [TG]NT |  |
|  | LYL | [GC]G[TC] |  |
|  |  |  |  |
| **GA11604** | RSL | [GC]TG | GNTNT[GT][GC]TG |
|  | VAT | NT[GT] |  |
|  | LER | GNT |  |
|  |  |  |  |
| **GA18503** | SIK | [TG]TT | [TG]TT |
|  |  |  |  |
| **GA20857** | GSR | GTT | NGG[GA]TTG[AT]TNNTGTT |
|  | SEG | NNT |  |
|  | NGR | G[AT]T |  |
|  | NIN | [GA]TT |  |
|  | RHT | NGG |  |
|  |  |  |  |
| **GA18174** | RTT | N[CT]G | NAGN[CT]G[TG]TCGA[GT]N[CT]G |
|  | VQR | GA[GT] |  |
|  | DSK | [TG]TC |  |
|  | YVV | N[CT]G |  |
|  | RNA | NAG |  |
|  |  |  |  |
| **GA20521** | HMA | NTN | NACNTN |
|  | DNS | NAC |  |
|  |  |  |  |
| **GA14502** | RYH | [GT]GG | [GT]NGN[AT]ANTN[GC]NTNTCNTTANTGT[GT]CNTNCT[GC]TTANNGNN[GA]TCA[AT]TNTT[CA][GA]GGTN[GT]GG |
|  | HSR | GTN |  |
|  | RHI | [CA][GA]G |  |
|  | TAS | NTT |  |
|  | NGQ | A[AT]T |  |
|  | DSN | [GA]TC |  |
|  | EER | GNN |  |
|  | KEQ | ANN |  |
|  | TAL | [GC]TT |  |
|  | SDV | NCT |  |
|  | SEF | CNT |  |
|  | VLR | GT[GT] |  |
|  | SEQ | ANT |  |
|  | SIE | NTT |  |
|  | DAA | NTC |  |
|  | SEL | [GC]NT |  |
|  | HKE | NTN |  |
|  | AGG | N[AT]A |  |
|  | FEH | [GT]NG |  |
|  |  |  |  |
| **GA11148** | DDR | GCC | [GA]ATNTTNTT[GT]TC[CA]ATNNTNTNNTTGCC |
|  | NSM | NTT |  |
|  | KLM | NTN |  |
|  | TEA | NNT |  |
|  | SNI | [CA]AT |  |
|  | DSK | [GT]TC |  |
|  | SAS | NTT |  |
|  | LAE | NTT |  |
|  | SNN | [GA]AT |  |
|  |  |  |  |
| **GA12282** | KHA | N[GA]N | [TG][GA]GGNGN[GA]N |
|  | RER | GNG |  |
|  | RHK | [TG][GA]G |  |
|  |  |  |  |
| **GA16779** | DAG | NTC | NTC |
|  |  |  |  |
| **GA12985** | GRR | GTT | G[CT]N[GT]TCNCTNNTNTANTTATNNTTGTCNTTGGNCTTGCCNTTGTT |
|  | TCE | NTT |  |
|  | DDR | GCC |  |
|  | SMF | CTT |  |
|  | EHR | GGN |  |
|  | TAE | NTT |  |
|  | DSR | GTC |  |
|  | GKA | NTT |  |
|  | ESQ | ATN |  |
|  | SRA | NTT |  |
|  | QSS | NTA |  |
|  | SEE | NNT |  |
|  | NDS | NCT |  |
|  | DAH | [GT]TC |  |
|  | EVR | G[CT]N |  |
|  |  |  |  |
| **GA14782** | DTQ | A[CT]C | A[CT]C |
|  |  |  |  |
| **GA11015** | LDG | NC[TC] | NTTGTA[TG][CT]C[CA]TG[TG][CT]TGT[GT]NTTNTCNC[TC] |
|  | DKV | NTC |  |
|  | NRA | NTT |  |
|  | VLR | GT[GT] |  |
|  | NTK | [TG][CT]T |  |
|  | RSI | [CA]TG |  |
|  | DVK | [TG][CT]C |  |
|  | QAR | GTA |  |
|  | NRA | NTT |  |
|  |  |  |  |
| **GA12301** | SCS | NTT | NTT |
|  |  |  |  |
| **GA18742** | GTE | N[CT]T | NTCNTTN[CT]T |
|  | GAA | NTT |  |
|  | DAM | NTC |  |
|  |  |  |  |
| **GA19446** | SKD | NTT | [TG]G[TC][TG]NTA[AT]GNTT |
|  | RGQ | A[AT]G |  |
|  | NEK | [TG]NT |  |
|  | LHK | [TG]G[TC] |  |
|  |  |  |  |
| **GA19399** | AVR | G[CT]A | G[CT]A |
|  |  |  |  |
| **GA17390** | YVN | [CT]GG | NNC GAANGG[CT]GG |
|  | RHT | NGG |  |
|  | QNR | GAA |  |
|  | DQS | NNC |  |
|  |  |  |  |
| **GA11272** | TSQ | ATT | NTTGNG[TG]NTATT |
|  | SEK | [TG]NT |  |
|  | FQR | GNG |  |
|  | SKA | NTT |  |
|  |  |  |  |
| **GA11802** | NNR | GAT | NCT[GC]TC[CA]TTNTGNGGGAT |
|  | RHE | NGG |  |
|  | RAA | NTG |  |
|  | SSI | [CA]TT |  |
|  | DLL | [GC]TC |  |
|  | NDA | NCT |  |
|  |  |  |  |
| **GA18080** | NDR | GCT | TA[CT]NNGGTTA[AT]TTTT[GA]TTATTNGTGCT |
|  | NHS | NGT |  |
|  | SAY | ATT |  |
|  | TKN | [GA]TT |  |
|  | TIK | TTT |  |
|  | GGQ | A[AT]T |  |
|  | NAR | GTT |  |
|  | REE | NNG |  |
|  | CNK | TA[CT] |  |
|  |  |  |  |
| **GA13472** | TLK | [TG][TC]T | NATN[CT]NNTTTTTGTTN[TC]TA[TC]N[GA]TC[TG][TC]T |
|  | DCN | [GA]TC |  |
|  | KLQ | A[TC]N |  |
|  | NLT | N[TC]T |  |
|  | GAR | GTT |  |
|  | NSK | TTT |  |
|  | NAS | NTT |  |
|  | KTE | N[CT]N |  |
|  | SNT | NAT |  |
|  |  |  |  |
| **GA22108** | KHA | N[GA]N | [GC][GA]GGNGN[GA]N |
|  | RER | GNG |  |
|  | RHL | [GC][GA]G |  |
|  |  |  |  |
| **GA14940** | TGY | A{AT]T | A[AT]T |
|  |  |  |  |
| **GA20818** | TVK | T[CT]T | GCTNTTT[CT]T |
|  | SLA | NTT |  |
|  | SDR | GCT |  |
|  | EDQ |  |  |
|  |  |  |  |
| **GA14083** | NQV | N[AT]T | [GC][CT]T[GA][CT]NNTTACTNT[CT]N[AT]T |
|  | CKE | NT[CT] |  |
|  | SDQ | ACT |  |
|  | SAV | NTT |  |
|  | ETN | [GA][CT]N |  |
|  | TVL | [GC][CT]T |  |
|  |  |  |  |
| **GA12650** | TLL | [GC]TT | N[AT]T[GC]TT |
|  | TQD | N[AT]T |  |
|  |  |  |  |
| **GA17791** | LLI | [CA]T[TC] | [TG]TTTTCNTTGG[TC]GTTG[AT]G[TG]CTGTG[TG]CT[CA]T[TC] |
|  | SDK | [TG]CT |  |
|  | RLR | GTG |  |
|  | SDK | [TG]CT |  |
|  | FGR | G[AT]G |  |
|  | TKR | GTT |  |
|  | LHR | GG[TC] |  |
|  | SSE | NTT |  |
|  | DAK | TTC |  |
|  | SRK | [TG]TT |  |
|  |  |  |  |
| **GA22115** | DAI | [CA]TC | NAN[CA]TC |
|  | KFV | NAN |  |
|  |  |  |  |
| **GA14042** | GNT | NAT | GTC[GA]NN[GT]TTNC[GT][TG]CGNAT |
|  | RDK | [TG]CG |  |
|  | VDV | NC[GT] |  |
|  | LLH | [GT]TT |  |
|  | KEN | [GA]NN |  |
|  | DSR | GTC |  |
|  |  |  |  |
| **GA14043** | TAA | NTT | NTN[TG]TTNCTNNANNGA[GA]GNTT |
|  | RHQ | A[GA]G |  |
|  | FQT | NNG |  |
|  | QQT | NNA |  |
|  | TDD | NCT |  |
|  | NAK | [TG]TT |  |
|  | ESV | NTN |  |
|  |  |  |  |
| **GA15842** | RQL | [GC]NG | [GA]ATN[GA]TNCTNNTATT[GC]NG |
|  | SSY | ATT |  |
|  | LQS | NNT |  |
|  | TDT | NCT |  |
|  | THS | N[GA]T |  |
|  | SNN | [GA]AT |  |
|  |  |  |  |
| **GA16514** | QQH | [GT][AT]A | A[AT]T[GT][AT]A |
|  | TGQ | A[AT]T |  |
|  |  |  |  |
| **GA13615** | CTA | N[CT]N | N[GA]N[GC]ACATGNTANGCAAAN[CT]N |
|  | QNY | AAA |  |
|  | DYS | NGC |  |
|  | QAM | NTA |  |
|  | RLQ | ATG |  |
|  | DNL | [GC]AC |  |
|  | KHT | N[GA]N |  |
|  |  |  |  |
| **GA15080** | SAS | NTT | NNT[GC][CT]GN[AT][CT][CA]AAGTNNA[GT]G[TG][GT]NTT |
|  | VRR | G[TG][GT] |  |
|  | VNV | NA[GT] |  |
|  | HKR | GTN |  |
|  | QQI | [CA]AA |  |
|  | CQV | N[AT][CT] |  |
|  | YVL | [GC][CT]G |  |
|  | NEA | NNT |  |
|  |  |  |  |
| **GA16381** | ANR | GAA | [TG]CGCAA[GA]AAN[CT]G G[CT]AGAA |
|  | QTR | G[CT]A |  |
|  | RTT | N[CT]G |  |
|  | QNN | [GA]AA |  |
|  | QNF | CAA |  |
|  | RDK | [TG]CG |  |
|  |  |  |  |
| **GA10314** | TGQ | A[AT]T | A[AT]GA[AT]TGTTA[AT]T |
|  | SRR | GTT |  |
|  | SGQ | A[AT]T |  |
|  | RGQ | A[AT]G |  |
|  |  |  |  |
| **GA10014** | KNI | [CA]AN | GCGNAAN[CT]ANGG[CA]AN |
|  | RHD | NGG |  |
|  | QTV | N[CT]A |  |
|  | QNT | NAA |  |
|  | RDR | GCG |  |
|  |  |  |  |
| **GA21126** | SVD | N[CT]T | N[CT]T |
|  |  |  |  |
| **GA17979** | QHL | [GC][GA]A | [GC][GA]A |
|  |  |  |  |
| **GA15455** | STA | N[CT]T | GAGN[AT]T[TG][TC]TNANN[CT]TGTNN[CT]T |
|  | KSR | GTN |  |
|  | NTA | N[CT]T |  |
|  | KNA | NAN |  |
|  | TLK | [TG][TC]T |  |
|  | TGT | N[AT]T |  |
|  | YNR | GAG |  |
|  |  |  |  |
| **GA12061** | SYT | NGT | N[AT]TNCNNGT |
|  | KDD | NCN |  |
|  | TGA | N[AT]T |  |
|  |  |  |  |
| **GA18499** | SAI | [CA]TT | NAT[CA]TTNTTATTNATNT[CT]NAT[CA]TT |
|  | TNV | NAT |  |
|  | CSM | NT[CT] |  |
|  | TNA | NAT |  |
|  | NIQ | ATT |  |
|  | SAS | NTT |  |
|  | SAI | [CA]TT |  |
|  | TNV | NAT |  |
|  |  |  |  |
| **GA18360** | RNR | GAG | GAGGAGGAGGAGGAGGANGAG |
|  | KNR | GAN |  |
|  | RDR | GAG |  |
|  | RNR | GAG |  |
|  | RNR | GAG |  |
|  | RNR | GAG |  |
|  | RNR | GAG |  |
|  |  |  |  |
| **GA13675** | TAF | CTT | CTT |
|  |  |  |  |
| **GA18538** | SRE | N[TG]T | [GA]TAC[CT]G[TG][CT]CG[AT]AN[TG]T |
|  | QQR | G[AT]A |  |
|  | DTK | [TG][CT]C |  |
|  | YTF | C[CT]G |  |
|  | QKN | [GA]TA |  |
|  |  |  |  |
| **GA21424** | YSS | NTG | [GC][AT]A[GA]ATNT[AG]N[GA]TNANNNT[GC]NTNTG |
|  | TEL | [GC]NT |  |
|  | SES | NNT |  |
|  | ENE | NAN |  |
|  | THA | N[GA]T |  |
|  | ASE | NT[AG] |  |
|  | SNN | [GA]AT |  |
|  | QGL | [GC][AT]A |  |
|  |  |  |  |
| **GA17156** | KSR | GTN | [CA]TTN[TC]T[CA]CAGTT[GC]CGG[CT]CGTN |
|  | RTR | G[CT]C |  |
|  | RDL | [GC]CG |  |
|  | SKR | GTT |  |
|  | QDI | [CA]CA |  |
|  | SLV | N[TC]T |  |
|  | SCI | [CA]TT |  |
|  |  |  |  |
| **GA12413** | AKQ | ATA | N[AT]N[TG]CGNTT[GC][AT]GGTT[TG]TT[GC]TAATA |
|  | AAL | [GC]TA |  |
|  | NAK | [TG]TT |  |
|  | MAR | GTT |  |
|  | RQL | [GC][AT]G |  |
|  | TAT | NTT |  |
|  | YDK | [TG]CG |  |
|  | HGA | N[AT]N |  |
|  |  |  |  |
| **GA26357** | DAE | NTC | N[TC]TNN[CT]GTTCANNTC |
|  | KDQ | CAN |  |
|  | SAR | GTT |  |
|  | CEE | NN[CT] |  |
|  | TLA | N[TC]T |  |
|  |  |  |  |
| **GA11300** | NNV | NAT | T[GA]ANAT |
|  | QHK | T[GA]A |  |
|  |  |  |  |
| **GA19755** | SHE | N[GA]T | NNTNCA[GA][CT][AG][TG]TTNNTAAAN[CT]T[TG]NTN[GA]T |
|  | SQK | [TG]NT |  |
|  | MTD | N[CT]T |  |
|  | QNQ | AAA |  |
|  | TES | NNT |  |
|  | TSK | [TG]TT |  |
|  | AVN | [GA][CT][AG] |  |
|  | QDM | NCA |  |
|  | SET | NNT |  |
|  |  |  |  |
| **GA19878** | SNE | NAT | [TG]TTNTCGNTNAT |
|  | TER | GNT |  |
|  | DSE | NTC |  |
|  | SCK | [TG]TT |  |
|  |  |  |  |
| **GA19875** | SKD | NTT | NNTN[CT]TT[CT]CNAANTT |
|  | ANT | NAA |  |
|  | DTK | T[CT]C |  |
|  | LTA | N[CT]T |  |
|  | LQA | NNT |  |
|  |  |  |  |
| **GA10205** | KRE | NTT | A[GA]T[TG]TGN[CT]G [TG]A[GT]NTT |
|  | VQK | [TG]A[GT] |  |
|  | YVA | N[CT]G |  |
|  | YSK | [TG]TG |  |
|  | LHQ | A[GA]T |  |
|  |  |  |  |
| **GA20743** | KST | NTN | NNCANTN[CT]A[TG][CT]C[CA]TT[GA][CT]CGTTN[GA][TC]GTNNTTGTGNTCNTGNNGNTN |
|  | RES | NNG |  |
|  | RAS | NTG |  |
|  | DSE | NTC |  |
|  | RLR | GTG |  |
|  | NAA | NTT |  |
|  | ESR | GTN |  |
|  | LHD | N[GA][TC] |  |
|  | NRR | GTT |  |
|  | DTN | [GA][CT]C |  |
|  | SSI | [CA]TT |  |
|  | DTK | [TG][CT]C |  |
|  | QVE | N[CT]A |  |
|  | SEQ | ANT |  |
|  | DQD | NNC |  |
|  |  |  |  |
| **GA21173** | TSR | GTT | [TG][CT]TNTTGATNAAG[CT][GT][TG]TA[CA][CT]TNAG[GC]TTGTT |
|  | SLL | [GC]TT |  |
|  | RNS | NAG |  |
|  | STI | [CA][CT]T |  |
|  | AAK | [TG]TA |  |
|  | VVR | G[CT][GT] |  |
|  | QQT | NAA |  |
|  | TQR | GAT |  |
|  | TLE | NTT |  |
|  | STK | [TG][CT]T |  |
|  |  |  |  |
| **GA21108** | HGR | G[AT]N | [TG]CA[GA]TGA[CT]TTCNAGANNNNTTNTCNACG[AT]N |
|  | DQE | NAC |  |
|  | DKV | NTC |  |
|  | TLE | NTT |  |
|  | HEE | NNN |  |
|  | QHQ | AGA |  |
|  | IDD | TCN |  |
|  | TVQ | A[CT]T |  |
|  | RAN | [GA]TG |  |
|  | QDK | [TG]CA |  |
|  |  |  |  |
| **GA10684** | TTV | N[CT]T | [GA]CGGAAGTAN[CT]T |
|  | QAR | GTA |  |
|  | ANR | GAA |  |
|  | RDN | [GA]CG |  |
|  |  |  |  |
| **GA16221** | GQL | [GC]AT | [GC]AT |
|  |  |  |  |
| **GA16212** | TLR | GTT | GTCGNNAAAG[CT]AGTNGTT |
|  | CSR | GTN |  |
|  | QVR | G[CT]A |  |
|  | QNQ | AAA |  |
|  | HGR | GNN |  |
|  | DAR | GTC |  |
|  |  |  |  |
| **GA18737** | RQD | NNG | NNG |
|  |  |  |  |
| **GA11363** | SDS | NCT | NCTA[AT]T |
|  | NGY | A[AT]T |  |
|  |  |  |  |
| **GA16227** | RNQ | AAG | GAGGTC[GC][CT]GAATN[CT][CT]AAG |
|  | CTD | N[CT][CT] |  |
|  | NNQ | AAT |  |
|  | RTL | [GC][CT]G |  |
|  | DAR | GTC |  |
|  | RNR | GAG |  |
|  |  |  |  |
| **GA18728** | HPN | [GA]TN | [GA]TN |
|  |  |  |  |
| **GA19851** | TVE | N[CT]T | TNC[GA]TTN[CT]T |
|  | SAN | [GA]TT |  |
|  | DEK | TNC |  |
|  |  |  |  |
| **fru** | RNA | NAG | NAG |
|  |  |  |  |
| **GA17500** | AVR | G[CT][AG] | TNTG[CT][AG] |
|  | SEK | TNT |  |
|  |  |  |  |
| **GA16329** | SEL | [GC]NT | [GC]NT |
|  |  |  |  |
| **GA15068** | DNL | [GC]AC | NGGG[AT]T[CA][CT]T[GC][TC]T[GC]AC |
|  | TLL | [GC][TC]T |  |
|  | NTI | [CA][CT]T |  |
|  | SGR | G[AT]T |  |
|  | RHA | NGG |  |
|  |  |  |  |
| **GA20726** | QTK | [TG][CT]A | [GA]AGN[CT]A[GC][TC]GGANNTTNTT[TG][CT]A |
|  | SSE | NTT |  |
|  | TAT | NTT |  |
|  | ENR | GAN |  |
|  | RLL | [GC][TC]G |  |
|  | QVS | N[CT]A |  |
|  | RQN | [GA]AG |  |
|  |  |  |  |
| **GA20969** | DSV | NTC | NTT[GC]TGA[CT]TTCGGTT[GT]NT[CA]NTT[CT]NN[GA]TATT[GT]ACNTC |
|  | DNH | [GT]AC |  |
|  | TSY | ATT |  |
|  | NHC | N[GA]T |  |
|  | HTW | T[CT]N |  |
|  | SEI | [CA]NT |  |
|  | SEH | [GT]NT |  |
|  | MAR | GTT |  |
|  | RDW | TCG |  |
|  | LVQ | A[CT]T |  |
|  | FSL | [GC]TG |  |
|  | TKT | NTT |  |
|  |  |  |  |
| **GA11304** | NLA | NTT | GTTNNNATTNTT |
|  | TIQ | ATT |  |
|  | EQD | NNN |  |
|  | LKR | GTT |  |
|  |  |  |  |
| **GA18168** | TRE | NTT | [GC]TNCAG[TG][CT]CGNANTT |
|  | QQR | GNA |  |
|  | DTK | [TG][CT]C |  |
|  | YNF | CAG |  |
|  | KKL | [GC]TN |  |
|  |  |  |  |
| **GA20291** | DND | NAC | NAN[TG][AT]TGTTN[AT]TNAC |
|  | TQM | N[AT]T |  |
|  | NSR | GTT |  |
|  | SGK | [TG][AT]T |  |
|  | EFG | NAN |  |
|  |  |  |  |
| **GA13577** | SCA | [GA]TT | [GA]TT |
|  |  |  |  |
| **GA10453** | TER | GNT | N[TC][GT]NATNNTGNT |
|  | TES | NNT |  |
|  | TQA | NAT |  |
|  | VLG | N[TC][GT] |  |
|  |  |  |  |
| **GA14887** | HLA | N[TC]N | GTC[GA][GA]A[GA][CT]G[GA][GA]A[GA]GNNTGN[TC]N |
|  | RSS | NTG |  |
|  | KYN | [GA]GN |  |
|  | QHN | [GA][GA]A |  |
|  | RTN | [GA][CT]G |  |
|  | QHN | [GA][GA]A |  |
|  | DAR | GTC |  |
|  |  |  |  |
| **GA11083** | DND | NAC | NAC |
|  |  |  |  |
| **GA12173** | MDY | ACT | GGNACT |
|  | EHR | GGN |  |
|  |  |  |  |
| **GA21187** | KLR | G[TC]N | GANGTAN[GA]T[GC][AT]C[GC]CGNTAGTCGTNGNT[GA][AT]TG[TC]N |
|  | SGN | [GA][AT]T |  |
|  | TER | GNT |  |
|  | EKR | GTN |  |
|  | DKR | GTC |  |
|  | QSA | NTA |  |
|  | RDL | [GC]CG |  |
|  | DQL | [GC][AT]C |  |
|  | SHS | N[GA]T |  |
|  | QLR | GTA |  |
|  | HNR | GAN |  |
|  |  |  |  |
| **GA15438** | CCK | [TG]T[CT] | [TG]T[CT] |
|  |  |  |  |
| **GA11715** | TNR | GAT | NACN[TC]GNTTGAT |
|  | SAM | NTT |  |
|  | RLG | N[TC]G |  |
|  | DNA | NAC |  |
|  |  |  |  |
| **GA13627** | LTS | N[CT][TC] | N[CT]TN[CT][TC] |
|  | NTE | N[CT]T |  |
|  |  |  |  |
| **GA18735** | NNS | NAT | N[TG]T[CA]TNGAGTTTGTNNAT |
|  | HKR | GTN |  |
|  | LRK | TTT |  |
|  | YNR | GAG |  |
|  | ISI | [CA]TN |  |
|  | TRV | N[TG]T |  |
|  |  |  |  |
| **GA15252** | DYG | NGC | GTCNTNGAANCTNGC |
|  | TDV | NCT |  |
|  | QQR | GAA |  |
|  | KSE | NTN |  |
|  | DKR | GTC |  |
|  |  |  |  |
| **GA15261** | VDL | [GC]C[GT] | N[AT]A[GT][AT]GN[AT]TN[CT]T[GA][GA]TGNTN[TC]GN[CT]TG[CT]N[GC]C[GT] |
|  | ETR | G[CT]N |  |
|  | TTV | N[CT]T |  |
|  | RLE | N[TC]G |  |
|  | TER | GNT |  |
|  | THN | [GA][GA]T |  |
|  | NTV | N[CT]T |  |
|  | SGE | N[AT]T |  |
|  | RGH | [GT][AT]G |  |
|  | QGG | N[AT]A |  |
|  |  |  |  |
| **GA29018** | SSS | NTT | N[AT]ANTGNCTN[CT]CNNTGAANCTNTT |
|  | TDG | NCT |  |
|  | QNR | GAA |  |
|  | TES | NNT |  |
|  | DVA | N[CT]C |  |
|  | SDV | NCT |  |
|  | RAV | NTG |  |
|  | QGA | N[AT]A |  |
|  |  |  |  |
| **GA16359-PB** | TTQ | A[CT]T | A[CT]T |
|  |  |  |  |
| **ttk** | RKN | [GA]TG | [GA]TG |
|  |  |  |  |
| **chinmo** | YNA | NAG | NAG |
|  |  |  |  |
| **cbt** | KHA | N[GA]N | [TG][GA]GGNGN[GA]N |
|  | RER | GNG |  |
|  | RHK | [TG][GA]G |  |
|  |  |  |  |
| **GA21140** | HTN | [GA][CT]N | [GA][CT]N |
|  |  |  |  |
| **GA17728** | KHA | N[GA]N | N[GA]N |
|  |  |  |  |
| **GA19930** | TPR | GTT | GTT |
|  |  |  |  |
| **GA21777** | TEQ | ANT | ANT |
|  |  |  |  |
| **GA13725** | SDI | [CA]CT | [CA]CT |
|  |  |  |  |
| **GA10988** | DER | GNC | NCCGNC |
|  | DDD | NCC |  |
|  |  |  |  |
| **GA17585** | CPP | [TG]T[CT] | GTN[TG]TG[TG]T[CT] |
|  | RSK | [TG]TG |  |
|  | KPR | GTN |  |
|  |  |  |  |
| **GA10182** | TEQ | ANT | [GA]TTN[CT]TNTGANT |
|  | RAG | NTG |  |
|  | SVE | N[CT]T |  |
|  | SKN | [GA]TT |  |
|  |  |  |  |
| **GA10264** | RNR | GAG | GAAGANGAG |
|  | INR | GAN |  |
|  | QNR | GAA |  |
|  |  |  |  |
| **GA13655** | INV | NAN | A[AT]A[GT]TTN[AT][AG]NAN |
|  | AQT | N[AT][AG] |  |
|  | SAH | [GT]TT |  |
|  | QGQ | A[AT]A |  |
|  |  |  |  |
| **GA16348** | TGV | N[AT]T | GCGNAA[TG]CAN[CT]GATNN[AT]T |
|  | HSQ | ATN |  |
|  | RTT | N[CT]G |  |
|  | QDK | [TG]CA |  |
|  | QNT | NAA |  |
|  | RDR | GCG |  |
|  |  |  |  |
| **GA11823** | DMQ | ATC | [TG]GGNA[CT]ATC |
|  | CNS | NA[CT] |  |
|  | RYK | [TG]GG |  |
|  |  |  |  |
| **GA15529** | KHA | N[GA]N | [TG][GA]GGNGN[GA]N |
|  | RER | GNG |  |
|  | RHK | [TG][GA]G |  |
|  |  |  |  |
| **GA15296** | YKQ | ATG | NAANTTATTGTTN[CT][GT]ATG |
|  | VTE | N[CT][GT] |  |
|  | SMR | GTT |  |
|  | SSY | ATT |  |
|  | SRS | NTT |  |
|  | AFE | NAA |  |
|  |  |  |  |
| **GA19865** | GDE | NCT | NTGGAA[GC]AGNTN[GA][AT][CT]NTTTNCNCT |
|  | DEK | TNC |  |
|  | TAV | NTT |  |
|  | WGN | [GA][AT][CT] |  |
|  | HAS | NTN |  |
|  | RNL | [GC]AG |  |
|  | QNR | GAA |  |
|  | RKE | NTG |  |
|  |  |  |  |
| **GA22104** | DTE | N[CT]C | N[TC]CA[AT]NN[CT]C |
|  | HGQ | A[AT]N |  |
|  | DLE | N[TC]C |  |
|  |  |  |  |
| **GA21024** | LHT | N[GA][TC] | A[AT]A[GC]TTANTN[CT]A[GA]NGN[GA]AN[GA][TC]N[GA][TC]NTNN[GA][TC] |
|  | HCT | NTN |  |
|  | LHT | N[GA][TC] |  |
|  | LHT | N[GA][TC] |  |
|  | QHM | N[GA]A |  |
|  | RQN | [GA]NG |  |
|  | QTS | N[CT]A |  |
|  | TEQ | ANT |  |
|  | SAL | [GC]TT |  |
|  | AGQ | A[AT]A |  |
|  |  |  |  |
| **GA10767** | LDI | [CA]CT | [CA]CT |
|  |  |  |  |
| **GA10912** | CQS | NNN | [GT][TC]TNNN |
|  | SLH | [GT][TC]T |  |
|  |  |  |  |
| **GA13915** | SCK | [TG]TT | [TG]AT[TG]TT |
|  | SFK | [TG]AT |  |
|  |  |  |  |
| **GA15299** | NEL | [GC]NT | [GA]AANCA[CA]AGNCGNGGNNN[TG]GTGTGN[GA]GGGGGGGNNN[GC]NT |
|  | HQA | NNN |  |
|  | RHR | GGG |  |
|  | RHR | GGG |  |
|  | RHA | N[GA]G |  |
|  | RAR | GTG |  |
|  | SYK | [TG]GT |  |
|  | EQE | NNN |  |
|  | RYV | NGG |  |
|  | RDV | NCG |  |
|  | RNI | [CA]AG |  |
|  | QDA | NCA |  |
|  | QNN | [GA]AA |  |
|  |  |  |  |
| **GA10924** | RNI | [CA]AG | [TG]TN[TG]CT[CA]AG |
|  | NDK | [TG]CT |  |
|  | HSK | [TG]TN |  |
|  |  |  |  |
| **GA21933** | SEA | NNT | NNT |
|  |  |  |  |
| **GA10863** | GDR | GCT | [GC][CT]NNNANCTNTTNATA[AT]G[GC]TT[GA]AAGCT |
|  | QNN | [GA]AA |  |
|  | LRL | [GC]TT |  |
|  | RQQ | A[AT]G |  |
|  | TNV | NAT |  |
|  | NAA | NTT |  |
|  | PDT | NCT |  |
|  | QQV | NNA |  |
|  | HVL | [GC][CT]N |  |
|  |  |  |  |
| **GA20900** | DEY | ANC | ANC |
|  |  |  |  |
| **GA22020** | NQG | N[AT]T | [TG][GA]GGNGN[AT]T |
|  | RER | GNG |  |
|  | RHK | [TG][GA]G |  |
|  |  |  |  |
| **GA14370** | TNR | GAT | NACN[TC]GNTTGAT |
|  | SAM | NTT |  |
|  | RLG | N[TC]G |  |
|  | DNA | NAC |  |
|  |  |  |  |
| **GA11205** | RLR | G[TC]G | NNTN[TG]GG[AT]C[TG][GA]GG[TC]G |
|  | RHK | [TG][GA]G |  |
|  | DGR | G[AT]C |  |
|  | RRT | N[TG]G |  |
|  | TEV | NNT |  |
|  |  |  |  |
| **GA21620** | GSS | NTT | NGNN[CT]G[TG]TCGNTNTGNTT |
|  | YAA | NTG |  |
|  | SER | GNT |  |
|  | DSK | [TG]TC |  |
|  | YVV | N[CT]G |  |
|  | KYA | NGN |  |
|  |  |  |  |
| **GA15499** | YEV | NNG | NATNAANTT[GC]TTGTGGTT[GA]TTNGTNNG |
|  | SYT | NGT |  |
|  | SRN | [GA]TT |  |
|  | NRR | GTT |  |
|  | FSR | GTG |  |
|  | NSL | [GC]TT |  |
|  | SAA | NTT |  |
|  | QNE | NAA |  |
|  | SNV | NAT |  |
|  |  |  |  |
| **GA20102** | WKQ | AT[CT] | ACG[TG]CG[TG][GA]TNTTGTTGATG[CT]CAT[CT] |
|  | DVR | G[CT]C |  |
|  | SFR | GAT |  |
|  | GSR | GTT |  |
|  | TAG | NTT |  |
|  | THK | [TG][GA]T |  |
|  | RDK | [TG]CG |  |
|  | RDQ | ACG |  |
|  |  |  |  |
| **GA10975** | SKA | NTT | A[GA]GNAG[TG]GNNAANTGNTT |
|  | RWD | NTG |  |
|  | QNA | NAA |  |
|  | HYK | [TG]GN |  |
|  | RNS | NAG |  |
|  | RHQ | A[GA]G |  |
|  |  |  |  |
| **GA11147** | SSV | NTT | [TG]ATN[AT][TG]NTT[TG]TCNAT[GC]CT[CA][TC]NNTT |
|  | KLI | [CA][TC]N |  |
|  | TDL | [GC]CT |  |
|  | TNV | NAT |  |
|  | DSK | [TG]TC |  |
|  | SKA | NTT |  |
|  | SGE | N[AT][TG] |  |
|  | NNK | [TG]AT |  |
|  |  |  |  |
| **GA21909** | RHR | GGG | [TG]ATAAG[GC]CN[TG]TNGTAGATGGNGGG |
|  | IHR | GGN |  |
|  | SNR | GAT |  |
|  | QKR | GTA |  |
|  | KIK | [TG]TN |  |
|  | KDL | [GC]CN |  |
|  | YNQ | AAG |  |
|  | SNK | [TG]AT |  |
|  |  |  |  |
| **GA10941** | QTQ | A[CT]A | [TG]NTA[TC]AA[TC]AA[GA]AA[CT]AA[TC]AA[GA]AA[CT]A |
|  | QHQ | A[GA]A |  |
|  | QIQ | A[TC]A |  |
|  | QTQ | A[CT]A |  |
|  | QHQ | A[GA]A |  |
|  | QIQ | A[TC]A |  |
|  | QIQ | A[TC]A |  |
|  | TEK | [TG]NT |  |
|  |  |  |  |
| **GA19031** | DGC | N[AT]C | N[AT]C |
|  |  |  |  |
| **GA17939** | NDR | GCT | GCT |
|  |  |  |  |
| **GA12616** | CNS | NA[CT] | [TG]GGNA[CT] |
|  | RYK | [TG]GG |  |
|  |  |  |  |
| **GA19534** | GRV | NTT | NTT |
|  |  |  |  |
| **GA21437** | HER | GNN | [TG]AG[TG]TTGNN |
|  | NRK | [TG]TT |  |
|  | RFK | [TG]AG |  |
|  |  |  |  |
| **GA21233** | QVD | N[CT]A | TTA[TG]ATN[TG]N[CA]AT[CA]NTNATN[AT][TA]N[CT]A |
|  | TQV | N[AT][TA] |  |
|  | MNV | NAT |  |
|  | TEI | [CA]NT |  |
|  | TFI | [CA]AT |  |
|  | ERD | N[TG]N |  |
|  | TNK | [TG]AT |  |
|  | QSW | TTA |  |
|  |  |  |  |
| **GA11559** | SAV | NTT | N[TC]T[CA]TAGTC[GC]TT[GA]NTA[CT]TNTT |
|  | STY | A[CT]T |  |
|  | NQN | [GA]NT |  |
|  | SSL | [GC]TT |  |
|  | DAR | GTC |  |
|  | QAI | [CA]TA |  |
|  | NLV | N[TC]T |  |
|  |  |  |  |
| **GA15463** | NAN | [GA]TT | NTT[GA]TT |
|  | SKA | NTT |  |
|  |  |  |  |
| **GA11840** | VGS | N[AT][GT] | N[AT][GT] |
|  |  |  |  |
| **GA10748** | VGS | N[AT][GT] | N[AT][GT] |
|  |  |  |  |
| **GA18980** | MNT | NAT | GNT[TG][GA]ANAT |
|  | QHK | [TG][GA]A |  |
|  | SER | GNT |  |
|  |  |  |  |
| **GA20315** | KKE | NTN | GTAATTGAT[TG]TG[CA]TN[GA]TT[TG]NT[GA]TN[TG]T[CT][CA][TC]G[GC]TTNTN |
|  | TAL | [GC]TT |  |
|  | RLI | [CA][TC]G |  |
|  | CAK | [TG]T[CT] |  |
|  | IAN | [GA]TN |  |
|  | TEK | [TG]NT |  |
|  | TAN | [GA]TT |  |
|  | KAI | [CA]TN |  |
|  | YSK | [TG]TG |  |
|  | NNR | GAT |  |
|  | NSQ | ATT |  |
|  | QAR | GTA |  |
|  |  |  |  |
| **GA14766** | SNE | NAT | [GA][AT]GNTTNNNNAT[TG]TTNTTNTTNCAN[CT]TNTTN[CT]CANTCTT[TG]ATN[TC]TNAT |
|  | GLM | N[TC]T |  |
|  | NQK | [TG]AT |  |
|  | SRF | CTT |  |
|  | NEQ | ANT |  |
|  | DVE | N[CT]C |  |
|  | TKS | NTT |  |
|  | STV | N[CT]T |  |
|  | QDS | NCA |  |
|  | NST | NTT |  |
|  | SSV | NTT |  |
|  | SAK | [TG]TT |  |
|  | NNT | NAT |  |
|  | HEA | NNN |  |
|  | NSV | NTT |  |
|  | RGN | [GA][AT]G |  |
|  |  |  |  |
| **GA17017** | ICA | [GA]TN | [GA]TN |
|  |  |  |  |
| **GA15581** | NEQ | ANT | NTG[GT][GA]TG[GA]GGNNC[TC]T[TG]TNG[CT][AG]N[GA]TG[TC]T[GA]TTNATACGN[TG]TN[TC]NANT |
|  | KLE | N[TC]N |  |
|  | TRS | N[TG]T |  |
|  | RDQ | ACG |  |
|  | TQV | NAT |  |
|  | SKN | [GA]TT |  |
|  | MLR | G[TC]T |  |
|  | MHA | N[GA]T |  |
|  | ATR | G[CT][AG] |  |
|  | EKK | [TG]TN |  |
|  | TLF | C[TC]T |  |
|  | HER | GNN |  |
|  | FHR | G[GA]G |  |
|  | THH | [GT][GA]T |  |
|  | RAV | NTG |  |
|  |  |  |  |
| **GA11925** | EQS | N[AT]N | [GC]TCATTN[TC]TNNTNAT[CA]TTNTCN[AT]N |
|  | DST | NTC |  |
|  | SKI | [CA]TT |  |
|  | SFV | NAT |  |
|  | NEV | NNT |  |
|  | NLE | N[TC]T |  |
|  | SRQ | ATT |  |
|  | DKL | [GC]TC |  |
|  |  |  |  |
| **GA14041** | SVS | N[CT]T | N[AT]AGTTNCN[GC][AT][TC][GC]C[GT][GA]NTN[CT]T |
|  | GEN | [GA]NT |  |
|  | VDL | [GC]C[GT] |  |
|  | LQL | [GC][AT][TC] |  |
|  | KDD | NCN |  |
|  | SSR | GTT |  |
|  | QGS | N[AT]A |  |
|  |  |  |  |
| **GA14054** | QTE | N[CT]A | N[CT]A |
|  |  |  |  |
| **GA18373** | NDR | GCT | [TG]ATNCGGTTA[AT]N[TG]ATNTTATT[CA][CT]CGCT |
|  | DTI | [CA][CT]C |  |
|  | TAY | ATT |  |
|  | TKE | NTT |  |
|  | SNK | [TG]AT |  |
|  | HGQ | A[AT]N |  |
|  | NAR | GTT |  |
|  | RDE | NCG |  |
|  | SNK | [TG]AT |  |
|  |  |  |  |
| **GA15849** | TSF | CTT | ATG[GA]TTN[AT]T[GT]A[TC][GC]T[GT][TG]NGNATCTT |
|  | NNM | NAT |  |
|  | REK | [TG]NG |  |
|  | VSL | [GC]T[GT] |  |
|  | LNH | [GT]A[TC] |  |
|  | TQA | N[AT]T |  |
|  | TAN | [GA]TT |  |
|  | RAQ | ATG |  |
|  |  |  |  |
| **GA10917** | RWA | NTG | NTG |
|  |  |  |  |
| **GA10271** | CCA | NT[CT] | NT[CT] |
|  |  |  |  |
| **GA16336** | SNQ | AAT | [TG]ATGTTG[GA]T[CA]TT[GA]AGACGATTGNTGTTG[AT]GA[TC]TGTTAAT |
|  | TAR | GTT |  |
|  | SLQ | A[TC]T |  |
|  | YQR | G[AT]G |  |
|  | SAR | GTT |  |
|  | TER | GNT |  |
|  | SAQ | ATT |  |
|  | YDY | ACG |  |
|  | RNN | [GA]AG |  |
|  | TSI | [CA]TT |  |
|  | SHR | G[GA]T |  |
|  | SKR | GTT |  |
|  | NNK | [TG]AT |  |
|  |  |  |  |
| **GA21630** | LEQ | AN[TC] | [GA]NAG[AT]GGCGNTG[GA]NT[TG]AG[TG]CGNTTAN[TC] |
|  | TAA | NTT |  |
|  | RDK | [TG]CG |  |
|  | RNK | [TG]AG |  |
|  | TEN | [GA]NT |  |
|  | RKA | NTG |  |
|  | RDR | GCG |  |
|  | RQR | G[AT]G |  |
|  | QEN | [GA]NA |  |
|  |  |  |  |
| **GA12497** | TAT | NTT | [GA]CT[TG]NTNTT |
|  | SEK | [TG]NT |  |
|  | SDN | [GA]CT |  |
|  |  |  |  |
| **GA10010** | KNI | [CA]AN | [CA]AN |
|  |  |  |  |
| **GA14721** | DIQ | A[TC]C | [CA][AT]TA[TC]C |
|  | TGI | [CA][AT]T |  |
|  |  |  |  |
| **GA21064** | TLL | [GC][TC]T | [GC][TC]T |
|  |  |  |  |
| **GA16882** | PPK | [TG]TT | NTT[TG]TT |
|  | GCC | NTT |  |
|  |  |  |  |
| **GA14528** | DFG | NAC | NAC |
|  |  |  |  |
| **GA17834** | SGK | [TG][AT]T | NA[CT]NTGN[AT][GT]NTT[GA]TTN[TC]TN[TG][GT][TG]TNANN[TG][AT]T |
|  | HEQ | ANN |  |
|  | IPK | [TG]TN |  |
|  | VRT | N[TG][GT] |  |
|  | SLE | N[TC]T |  |
|  | GSN | [GA]TT |  |
|  | TIT | NTT |  |
|  | VQS | N[AT][GT] |  |
|  | YST | NTG |  |
|  | CNA | NA[CT] |  |
|  |  |  |  |
| **GA17430** | TDA | NCT | GC[GT]NG[AG][GC]TGACANTTATTNCT |
|  | SAY | ATT |  |
|  | NAA | NTT |  |
|  | QDY | ACA |  |
|  | RSL | [GC]TG |  |
|  | AYS | NG[AG] |  |
|  | VDR | GC[GT] |  |
|  |  |  |  |
| **GA11281** | RHE | N[GA]G | NTA[TG]G[GT]A[CT]AN[CT]TNCTN[GA]G |
|  | SDE | NCT |  |
|  | TTM | N[CT]T |  |
|  | QTQ | A[CT]A |  |
|  | VYK | [TG]G[GT] |  |
|  | QAV | NTA |  |
|  |  |  |  |
| **GA17171** | HSN | [GA]TN | [GA]TN |
|  |  |  |  |
| **GA15759** | HTA | N[CT]N | [GA]TA[GT][CT]TNNGATNA[CT]TAAA[TG]TTN[AT]GN[CT]N |
|  | FGT | N[AT]G |  |
|  | SAK | [TG]TT |  |
|  | QNY | AAA |  |
|  | STY | A[CT]T |  |
|  | KMQ | ATN |  |
|  | REA | NNG |  |
|  | STH | [GT][CT]T |  |
|  | QCN | [GA]TA |  |
|  |  |  |  |
| **GA19783** | MNI | [CA]AT | NAGNTAGTNTTT[TG]TG[CA]TC[CA]AT |
|  | DMI | [CA]TC |  |
|  | YKK | [TG]TG |  |
|  | SAW | TTT |  |
|  | EKR | GTN |  |
|  | QKA | NTA |  |
|  | YNT | NAG |  |
|  |  |  |  |
| **GA19554** | NEF | CNT | [TG][AT]ANCTCNT |
|  | TDG | NCT |  |
|  | QQK | [TG][AT]A |  |
|  |  |  |  |
| **GA18481** | EKV | NTN | N[GA]AN[CT]G[TG][CT]CGTANTN |
|  | QSR | GTA |  |
|  | DTK | [TG][CT]C |  |
|  | YVG | N[CT]G |  |
|  | QHA | N[GA]A |  |
|  |  |  |  |
| **GA22043** | GAL | [GC]T[TG] | N[GA][TC][GA]TT[TG][CT]CGNT[GC]T[TG] |
|  | TER | GNT |  |
|  | DTK | [TG][CT]C |  |
|  | TIN | [GA]TT |  |
|  | LHT | N[GA][TC] |  |
|  |  |  |  |
| **hb** | TDA | NCT | N[AT][TG][CA][TC]C[GC]TGNTTA[GA]GNCT |
|  | FHY | A[GA]G |  |
|  | NMS | NTT |  |
|  | YSL | [GC]TG |  |
|  | DLI | [CA][TC]C |  |
|  | GGV | N[AT][TG] |  |
|  |  |  |  |
| **GA10237** | KHQ | A[GA]N | [TG][GA]T[TG]AGG[GA]GA[GA]N |
|  | RHR | G[GA]G |  |
|  | RNK | [TG]AG |  |
|  | THK | [TG][GA]T |  |
|  |  |  |  |
| **GA11270** | WDR | GC[CT] | [GA]TA[GC]CAATGNCG[CA]ATN[TG]G[GC]A[CT][GT][CT]TANGAAGGCT[GC]A[CT]NNG[GA]CNN[AT]GGC[CT]AA[CT]ATGNNGCTGGC[CT] |
|  | RKF | CTG |  |
|  | RES | NNG |  |
|  | RKQ | ATG |  |
|  | WNQ | AA[CT] |  |
|  | WDR | GC[CT] |  |
|  | RGS | N[AT]G |  |
|  | KDN | [GA]CN |  |
|  | RQA | NNG |  |
|  | WNL | [GC]A[CT] |  |
|  | MDR | GCT |  |
|  | RNQ | AAG |  |
|  | REQ | ANG |  |
|  | SLH | [GT][CT]T |  |
|  | WNL | [GC]A[CT] |  |
|  | RRA | N[TG]G |  |
|  | SNI | [CA]AT |  |
|  | RDC | NCG |  |
|  | RKQ | ATG |  |
|  | QDL | [GC]CA |  |
|  | QAN | [GA]TA |  |
|  |  |  |  |
| **GA12410** | SAI | [CA]TT | [CA]TT |
|  |  |  |  |
| **GA15242** | SDM | NCT | N[AT]T[TG]CTNCT |
|  | NDK | [TG]CT |  |
|  | SQS | N[AT]T |  |
|  |  |  |  |
| **GA26473** | NSE | NTT | NTT |
|  |  |  |  |
| **GA26478** | SAR | GTT | NTTGTT |
|  | TST | NTT |  |
|  |  |  |  |
| **GA21104** | LLM | N[TC][TC] | [GA]NTN[TC][TC] |
|  | SEN | [GA]NT |  |
|  |  |  |  |
| **GA14725** | CNV | NA[CT] | N[GA]GNT[GT]A[AT]TG[TC]TNA[CT] |
|  | TLR | G[TC]T |  |
|  | NGQ | A[AT]T |  |
|  | VKA | NT[GT] |  |
|  | RHA | N[GA]G |  |
|  |  |  |  |
| **GA14071** | SLE | N[TC]T | GTTNCTN[TC]T |
|  | NDA | NCT |  |
|  | NAR | GTT |  |
|  |  |  |  |
| **GA18142** | DSV | NTC | [TG][GA]ANTC |
|  | QHK | [TG][GA]A |  |
|  |  |  |  |
| **GA12131** | ESE | NTN | ATNN[GA]NGTNGTTANNNTN |
|  | SQQ | ANN |  |
|  | NRR | GTT |  |
|  | KSR | GTN |  |
|  | HHE | N[GA]N |  |
|  | HSQ | ATN |  |
|  |  |  |  |
| **GA13064** | MGK | [TG][AT]T | [TG]ATATTNTTG[GA]N[GT]AA[TG][AT]T |
|  | QNH | [GT]AA |  |
|  | EHR | G[GA]N |  |
|  | TCV | NTT |  |
|  | SKQ | ATT |  |
|  | NNK | [TG]AT |  |
|  |  |  |  |
| **GA18349** | HAE | NTN | [TG]TC[TG]CT[CA]AGNTN |
|  | RNI | [CA]AG |  |
|  | NDK | [TG]CT |  |
|  | DSK | [TG]TC |  |
|  |  |  |  |
| **GA16105** | AND | NA[AG] | [TG]ATNGGGAG[GA]CG[CA]ATGTNAAGNT[GT][GC]AGN[TC]TNCNNA[AG] |
|  | KDA | NCN |  |
|  | SLA | N[TC]T |  |
|  | RNL | [GC]AG |  |
|  | VAE | NT[GT] |  |
|  | RNQ | AAG |  |
|  | HSR | GTN |  |
|  | TNI | [CA]AT |  |
|  | RDN | [GA]CG |  |
|  | RNR | GAG |  |
|  | RYE | NGG |  |
|  | TNK | [TG]AT |  |
|  |  |  |  |
| **GA20515** | RTT | N[CT]G | GAA[TG][CT]CNTANAAN[CT]G |
|  | QNA | NAA |  |
|  | QST | NTA |  |
|  | DTK | [TG][CT]C |  |
|  | QNR | GAA |  |
|  |  |  |  |
| **GA27328** | DSK | [TG]TC | [TG]TC |
|  |  |  |  |
| **GA16175** | RET | NNG | NNG |
|  |  |  |  |
| **GA13193** | KAI | [CA]TN | ANG[GA][CT]GGTCGN[TG][CA]TN |
|  | GER | GN[TG] |  |
|  | DSR | GTC |  |
|  | YVN | [GA][CT]G |  |
|  | RQQ | ANG |  |
|  |  |  |  |
| **GA13150** | GKI | [CA]TT | NTA[GA][CT]N[TG]TG[TG]ATNTTN[TC]T[CA]TT |
|  | NLM | N[TC]T |  |
|  | MSV | NTT |  |
|  | TFK | [TG]AT |  |
|  | FSK | [TG]TG |  |
|  | ETN | [GA][CT]N |  |
|  | QSV | NTA |  |
|  |  |  |  |
| **GA22028** | TDK | [TG]CT | NTC[TG]CT |
|  | DCA | NTC |  |
|  |  |  |  |
| **GA19962** | DLQ | A[TC]C | [TG][GA]G[GC]NGG[GA]GA[TC]C |
|  | RHR | G[GA]G |  |
|  | RQL | [GC]NG |  |
|  | RHK | [TG][GA]G |  |
|  |  |  |  |
| **GA14950** | RYR | GGG | [TG][CT]A[GC]CAGTGNNGNTT[GA][TG]T[TG]A[CT] ANG[TG]GGNTNNANNNGGCNNTTGGG |
|  | SAM | NTT |  |
|  | KDR | GCN |  |
|  | RQD | NNG |  |
|  | WNA | NAN |  |
|  | KSV | NTN |  |
|  | RYK | [TG]GG |  |
|  | REQ | ANG |  |
|  | WNK | [TG]A[CT] |  |
|  | MRN | [GA][TG]T |  |
|  | SAT | NTT |  |
|  | RES | NNG |  |
|  | RKR | GTG |  |
|  | QDL | [GC]CA |  |
|  | QTK | [TG][CT]A |  |
|  |  |  |  |
| **GA14373** | RDR | GCG | GTT[GC]TGGTNGCG |
|  | KCR | GTN |  |
|  | RSL | [GC]TG |  |
|  | TSR | GTT |  |
|  |  |  |  |
| **GA20848** | NSR | GTT | N[GA][TC]ATG[TG][AT]TNNTGTT |
|  | SEA | NNT |  |
|  | SGK | [TG][AT]T |  |
|  | RIQ | ATG |  |
|  | LHT | N[GA][TC] |  |
|  |  |  |  |
| **GA11182** | SVR | G[CT]T | N[GA]G[GA]TT[TG][AT]TNNTG[CT]T |
|  | TES | NNT |  |
|  | SGK | [TG][AT]T |  |
|  | NIN | [GA]TT |  |
|  | RHT | N[GA]G |  |
|  |  |  |  |
| **GA27525** | ETR | G[CT]N | N[CT]TG[CT]N |
|  | STE | N[CT]T |  |
|  |  |  |  |
| **GA18456** | DNV | NAC | AAGG[TC]TNAC |
|  | TLR | G[TC]T |  |
|  | RNQ | AAG |  |
|  |  |  |  |
| **GA18968** | NYQ | AGT | [TG]GAN[CT]CNAAA[GA]AAGT |
|  | QHQ | A[GA]A |  |
|  | QNS | NAA |  |
|  | DTE | N[CT]C |  |
|  | QYK | [TG]GA |  |
|  |  |  |  |
| **GA18222** | SNR | GAT | NTNNCC[TG][CT]NGAT[TG]CNGAT |
|  | EDK | [TG]CN |  |
|  | TNR | GAT |  |
|  | ETK | [TG][CT]N |  |
|  | DDS | NCC |  |
|  | EAA | NTN |  |
|  |  |  |  |
| **GA10328** | TEA | NNT | N[CT]NN[AT]T[CA][AT]TGTAN[TG][TG]N[TC]TNNT |
|  | TLE | N[TC]T |  |
|  | GRS | N[TG][TG] |  |
|  | QAR | GTA |  |
|  | TGI | [CA][AT]T |  |
|  | TQV | N[AT]T |  |
|  | HTS | N[CT]N |  |
|  |  |  |  |
| **GA10277** | RTR | G[CT]G | NAT[GA]TGG[CT]T[GT]TTNATATTGNNG[CT]G |
|  | TQR | GNN |  |
|  | SSQ | ATT |  |
|  | SNV | NAT |  |
|  | SRH | [GT]TT |  |
|  | STR | G[CT]T |  |
|  | RAN | [GA]TG |  |
|  | NNT | NAT |  |
|  |  |  |  |
| **GA13430** | EAK | [TG]TN | [TG]ANATN[GC]T[AG][TG]TN |
|  | AAL | [GC]T[AG] |  |
|  | KSQ | ATN |  |
|  | KNK | [TG]AN |  |
|  |  |  |  |
| **GA13387** | LDR | GCT | N[AT]TGCT |
|  | SGT | N[AT]T |  |
|  |  |  |  |
| **GA14480** | KQL | [GC]NN | [GC]NN |
|  |  |  |  |
| **GA17456** | NAK | [TG]TT | NAANA[TC]GTN[TG]TT |
|  | EKR | GTN |  |
|  | LNT | NA[TC] |  |
|  | QNS | NAA |  |
|  |  |  |  |
| **GA17655** | TNT | NAT | NTTN[GA][AG][TG]CTN[TG]T[CA]TTNAT |
|  | TSI | [CA]TT |  |
|  | TRA | N[TG]T |  |
|  | TDK | [TG]CT |  |
|  | AHT | N[GA][AG] |  |
|  | TSS | NTT |  |
|  |  |  |  |
| **GA16697** | NNV | NAT | [TG]TTNAT |
|  | SAK | [TG]TT |  |
|  |  |  |  |
| **GA10519** | KIE | NTN | NTN |
|  |  |  |  |
| **GA14687** | GNT | NAT | NTTGAT[TG]CGN[AT]TN[TC]TN[TG]TNNTNAT |
|  | SED | NNT |  |
|  | NRE | N[TG]T |  |
|  | TLS | N[TC]T |  |
|  | SGT | N[AT]T |  |
|  | RDK | [TG]CG |  |
|  | TQR | GAT |  |
|  | SST | NTT |  |
|  |  |  |  |
| **GA17666** | TGK | [TG][AT]T | NACN[TC]GNTT[TG][AT]T |
|  | SAM | NTT |  |
|  | RLG | N[TC]G |  |
|  | DNA | NAC |  |
|  |  |  |  |
| **GA22134** | NYT | NGT | NNTANN[GC][TC]NN[GA]NA[AT]TG[CT][CT]NTTN[TG]TNTNGAG[TG]TNNNGGTNNGT |
|  | HKR | GTN |  |
|  | YQA | NNG |  |
|  | IRK | [TG]TN |  |
|  | YNR | GAG |  |
|  | ISV | NTN |  |
|  | TRV | N[TG]T |  |
|  | NAA | NTT |  |
|  | WVR | G[CT][CT] |  |
|  | SQY | A[AT]T |  |
|  | KHS | N[GA]N |  |
|  | ELL | [GC][TC]N |  |
|  | KEQ | ANN |  |
|  | TEE | NNT |  |
|  |  |  |  |
| **GA17803** | TGK | [TG][AT]T | NACN[TC]GNTT[TG][AT]T |
|  | TAM | NTT |  |
|  | RLG | N[TC]G |  |
|  | DNA | NAC |  |
|  |  |  |  |
| **GA17994** | TGK | [TG][AT]T | [TG][TC]GNACN[TC]GNTT[TG][AT]T[TG][AT]T |
|  | TGK | [TG][AT]T |  |
|  | SAM | NTT |  |
|  | RLG | N[TC]G |  |
|  | DNA | NAC |  |
|  | RLK | [TG][TC]G |  |
|  |  |  |  |
| **GA14322** | TND | NAT | ATNNNNA[GA]TNAT |
|  | SHQ | A[GA]T |  |
|  | SQM | NNN |  |
|  | HSQ | ATN |  |
|  |  |  |  |
| **GA10668** | SDR | GCT | NNA[GT]TGN[TC]TNTGNATG[CT]TNT[GT]NGTGCT |
|  | SYT | NGT |  |
|  | VAA | NT[GT] |  |
|  | NTR | G[CT]T |  |
|  | NNS | NAT |  |
|  | YAV | NTG |  |
|  | SLE | N[TC]T |  |
|  | RAH | [GT]TG |  |
|  | QGA | NNA |  |
|  |  |  |  |
| **GA26228** | DYI | [CA]GC | [CA]GN[CA]GC |
|  | EYI | [CA]GN |  |

**Supplementary Table 1**: *D. pseudoobscura* predicted zinc finger motifs. The gene name is listed in column one. In column two, each row represents a zinc finger within that particular protein. The amino acids at positions -1, 3, and 6 are indicated with their corresponding predicted nucleotide binding motif (column 3). The whole motif is listed in column four in the orientation it would be found in the sequence.
